# A novel multifunctional microneedle patch for synergistic photothermal- gas therapy against maxillofacial malignant melanoma and associated skin defects

**DOI:** 10.1186/s12951-024-02409-4

**Published:** 2024-04-23

**Authors:** Shaojie Dong, Yuwei Zhang, Yifei Zhang, Yukun Mei, Ahmadi Sina, Rui Zou, Lin Niu

**Affiliations:** 1 Key Laboratory of Shaanxi Province for Craniofacial Precision Medicine Research, College of Stomatology, Xi’an Jiaotong University, Xi’an, 710004 Shaanxi Province China; 2 Clinical Research Center of Shaanxi Province for Dental and Maxillofacial Diseases, Xi’an, 710004 Shaanxi Province China; 3 Department of Prosthodontics, College of Stomatology, Xi’an Jiaotong University, Xi’an, 710004 Shaanxi Province China

**Keywords:** Maxillofacial malignant melanoma, Microneedle, Photothermal therapy, Gas therapy, Skin defect

## Abstract

Considering the high recrudescence and the long-lasting unhealed large-sized wound that affect the aesthetics and cause dysfunction after resection of maxillofacial malignant skin tumors, a groundbreaking strategy is urgently needed. Photothermal therapy (PTT), which has become a complementary treatment of tumors, however, is powerless in tissue defect regeneration. Therefore, a novel multifunctional sodium nitroprusside and Fe^2+^ ions loaded microneedles (SNP-Fe@MNs) platform was fabricated by accomplishing desirable NIR-responsive photothermal effect while burst releasing nitric oxide (NO) after the ultraviolet radiation for the ablation of melanoma. Moreover, the steady releasing of NO in the long term by the platform can exert its angiogenic effects via upregulating multiple related pathways to promote tissue regeneration. Thus, the therapeutic dilemma caused by postoperative maxillofacial skin malignancies could be conquered through promoting tumor cell apoptosis via synergistic PTT—gas therapy and subsequent regeneration process in one step. The bio-application of SNP-Fe@MNs could be further popularized based on its ideal bioactivity and appealing features as a strategy for synergistic therapy of other tumors occurred in skin.

**Graphical Abstract:**

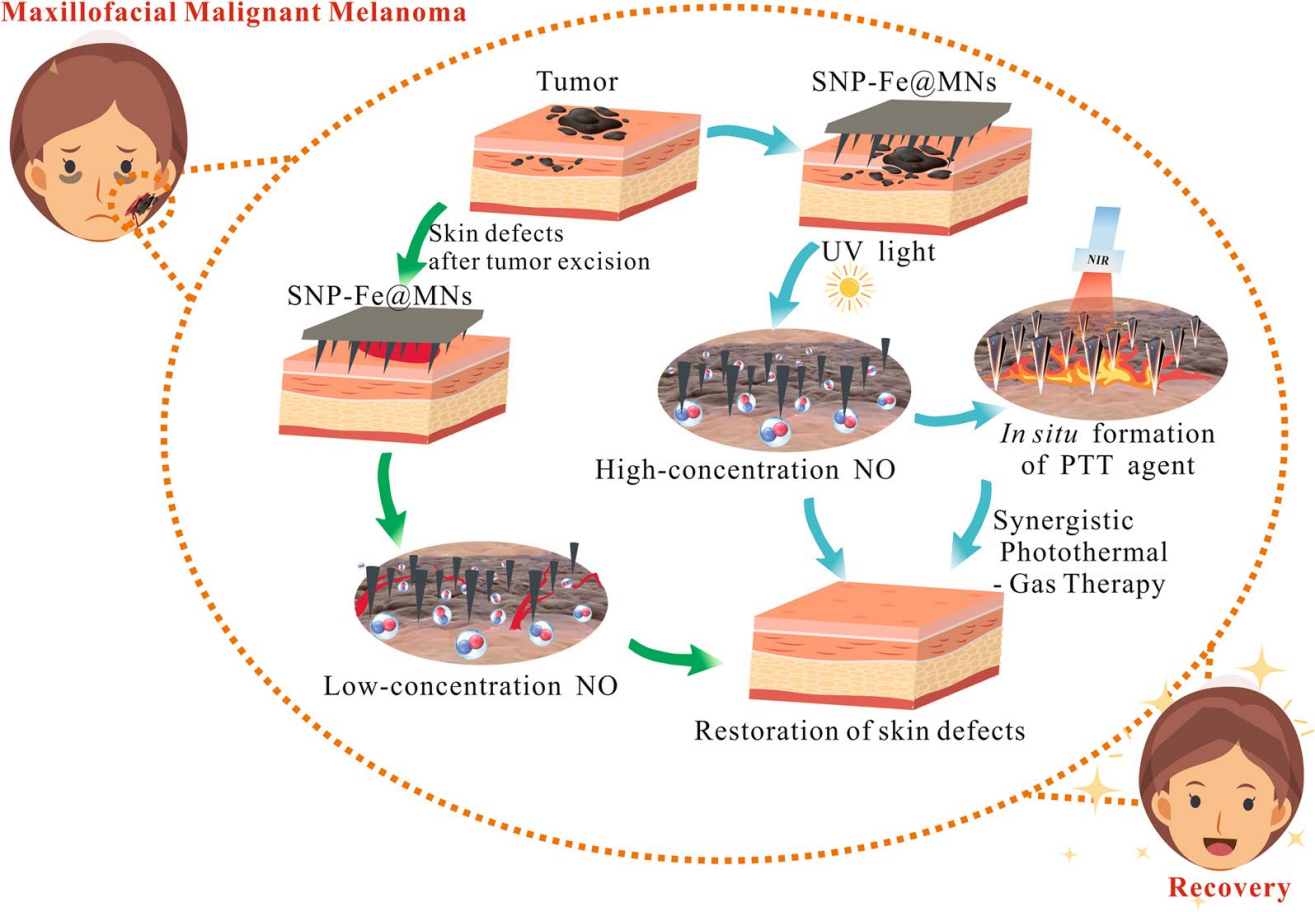

**Supplementary Information:**

The online version contains supplementary material available at 10.1186/s12951-024-02409-4.

## Introduction

Malignancies that derive from keratinocytes or melanocytes constitute the most commonly diagnosed tumors in skin [1]. With significantly increasing numbers over the past 50 years, at least seventy-six thousand cases of cutaneous melanoma are diagnosed each year [2, 3]. Meanwhile, 18–22% of new melanoma cases occurred in the head and neck region, as estimated [4–7]. As one of the most aggressive and deadliest forms of skin malignancies, malignant melanoma in maxillofacial region usually involved to the cheek and occipital scalp, which might cause severe dysfunction and psychosocial aesthetic disorder, making melanoma be hardly cured through traditional therapeutic approaches [6]. Classic surgical extensive resection regime of melanoma could merely be successfully in the nonmetastatic primary stage to prolong the survival rate of patients. However, it was limited by the difficulty of complete resection in maxillofacial region. Additionally, the high recurrence and metastasis rate often accompany the post operation period. Due to multi-drug resistance, poor respond with a subset of patients of immune checkpoint inhibitor, strict indications, a complex course of disease, and ineluctable side effects, consequently, making chemotherapy, radiotherapy, immunotherapy, and other conventional adjuvant therapies have low survival rate of patients. Moreover, the skin defect after excision in clinical practice can hardly self-cure on account of the huge lesion area or prophylactic expanded resection, resulting in severe dysfunction and aesthetic problems [8–10]. Therefore, a treatment strategy that can effectively induce the ablation of the residual invasive tumor cells and promote wound healing is essential for prolonging the survival rate of patients with maxillofacial malignant skin tumors.

For this reason, alternative therapeutic strategies were proposed over the recent years. Photothermal therapy (PTT), one of the photosensitizer-based and light-activated therapies, was based on the conception that heat-sensitivity of tumor cells is higher than that of normal somatic cells [11, 12]. As a rather safe modality of tumor ablation strategy, PTT could be utilized for the ablation of residual tumor cells after surgery [13–15]. However, because of nonuniform heat distribution and inevitably factors including skin barrier, deep position, and oversized volume of tumor, PTT often confront the issue that the temperature away from laser spot is insufficient to reach the threshold to induce apoptosis of tumor cells [16]. Furthermore, hyperthermia generated during the *in-situ* ablation process could result in overheating, which would induce irreversible burn damage that led to necrosis in the epidermis, dermis, and subcutaneous tissues of the surrounding skin [17, 18]. Occasionally, PTT suppressed primary tumor via thermal ablation but failed to achieve complete eradication to prevent recurrence and metastasis [19]. Therefore, a cooperative therapeutic strategy along with photothermal to ensure postoperative efficacy of malignant melanoma are needed.

As a short-lived, endogenously small gaseous signaling molecule, nitric oxide (NO) participates in regulating apoptotic processes, mitochondrial function, and other multiple pathways. Nevertheless, the effect of NO in tumor biology still remains controversial. Notably, NO has been proved to own both anti-tumorigenic and carcinogenic roles, which depends on the concentration, location, and time of duration. The parallel double roles of NO were also confirmed in melanoma, where NO mediates its effects through the formation of free radicals and enzymatic processes. Intriguingly, NO may aid in escaping from immune surveillance and promoting tumor proliferation by promoting tumor angiogenesis, invasion, and metastasis while inhibiting apoptosis at a rather low concentration. However, a reversal of the situation happens when NO can also mediate the regression of melanoma tumor in certain circumstances, basically a rather high concentration. It has been confirmed that immune cells may facilitate the generation of high levels of NO by macrophages to induce the apoptosis of B16 murine malignant melanoma cells via the most common caspase—3 pathways of apoptosis [20–22]. Noteworthily, NO can also dedicate to wound healing under a certain level to accelerate tissue regeneration through vascularization, which is critical for the recovery of post operation. Researches proved that NO exert its angiogenic effects via multiple pathways [23, 24]. Therefore, it is necessary to utilize NO in a beneficial way to avoid harm. By taking advantage of its gas therapeutic efficacy at high concentrations and its advantages in promoting the regeneration of maxillofacial defects at low concentrations, it is necessary to design a reasonable controlled release platform to carry drugs to achieve precise medical treatment.

Sodium nitroprusside (SNP), with the similar chemical structures with potassium ferricyanide, could be treated as a laser-controlled nitric oxide (NO) release platform via allowing SNP to participate in the formation of mesoporous Prussian blue nanoparticles. Subsequent findings enlightened that the product formed by SNP coordinating with Ferrous Lactate could be treated as a new photothermal initiator [25]. SNP could be activated by the ultraviolet or natural light and release NO, meanwhile, combined with the gradually oxidized Fe^2+^ and finally form a Prussian blue photothermal product in situ.

Thus, the combination of SNP and Fe^2+^ could be treated as a novel donor of NO gas. The burst release of NO can contribute as gas therapeutic agents toward tumors. In addition, the photothermal potential of the products from the decomposition reaction of SNP under the irradiation of near infrared (NIR) lasers can be utilized for PTT as a synergistic therapy to overcome the disappointing current treatments of malignant melanoma. What must be distinguished is the timing and targeted concentration determine whether NO exerts which function. Thus, a controlled NO delivery route is necessary for the burst releasing of NO at the early ablation stage and the gradual, steady releasing of NO in the regeneration phase.

**Fig. 1:**
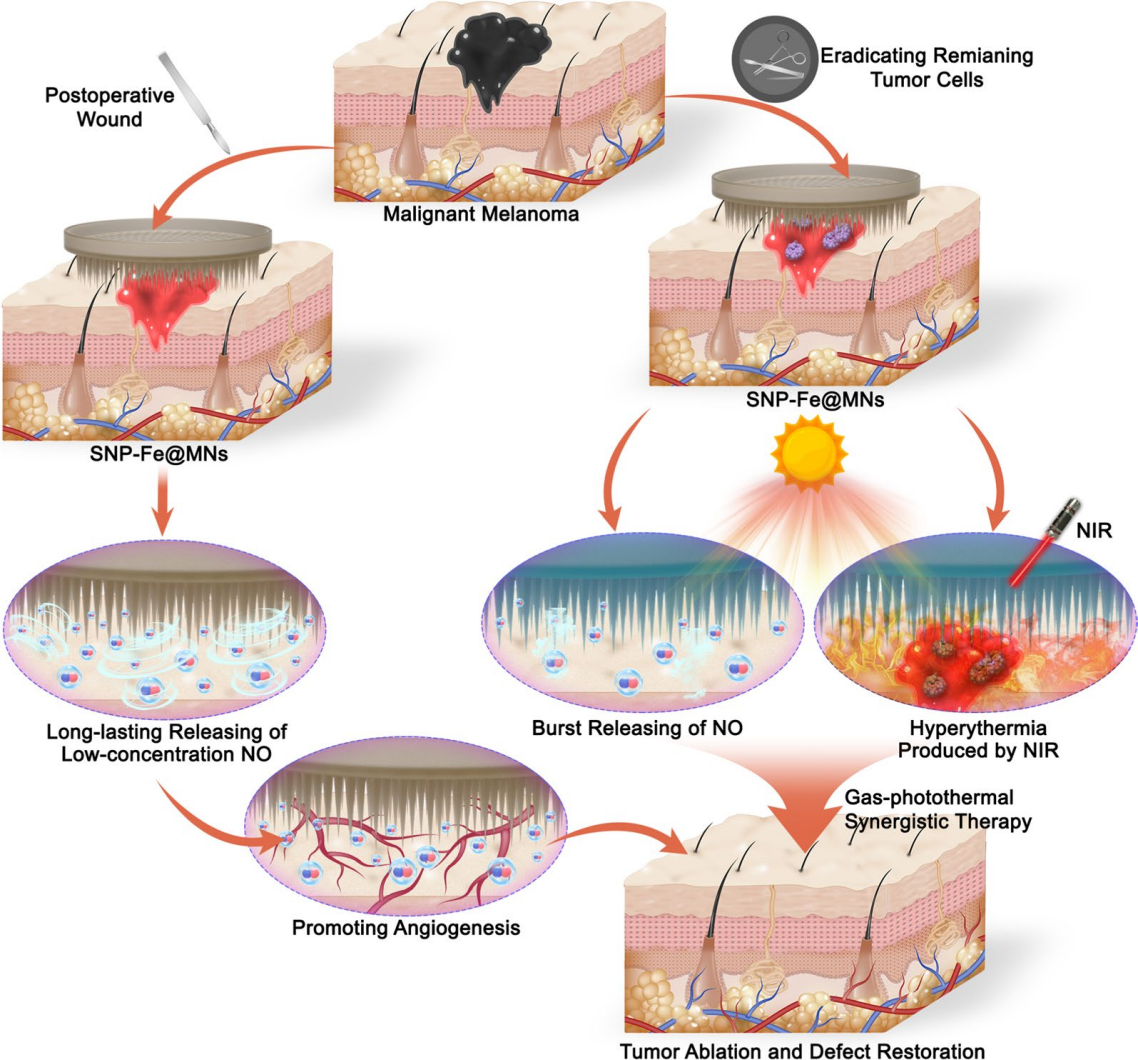
Schematic therapeutic procedures of the sodium nitroprusside and Fe^2+^ ions loaded microneedles SNP-Fe@MNs treating malignant melanoma in the head and neck region

Microneedles (MNs), one of the effective minimally invasive delivery platforms, can be utilized to puncture through the cuticle to the dermis with the minimal loss to deliver agents to the targeted location of malignant skin tumor [26]. Additionally, MNs have obvious superiorities over other routes of administration, including non-pain, readily-permitted transport of drugs, simplicity, good repeatability, ideal organizational integration, biosafety and developed clinical applications, which have been collaborated with multiple therapy strategies against cancers [27, 28]. As a consequence, sodium nitroprusside and Fe^2+^ ions loaded microneedles (SNP-Fe@MNs) was fabricated and displays broad prospects as a novel and alternative therapeutic strategy against malignant melanoma in the head and neck region by preventing recurrence and metastasis of tumor and promoting early wound healing, which would be conducive to the restoration of latent facial deformities and the compensation for serious physical, psychosocial, and cultural consequences (Fig. 1).

## Results and discussion

**Fig. 2:**
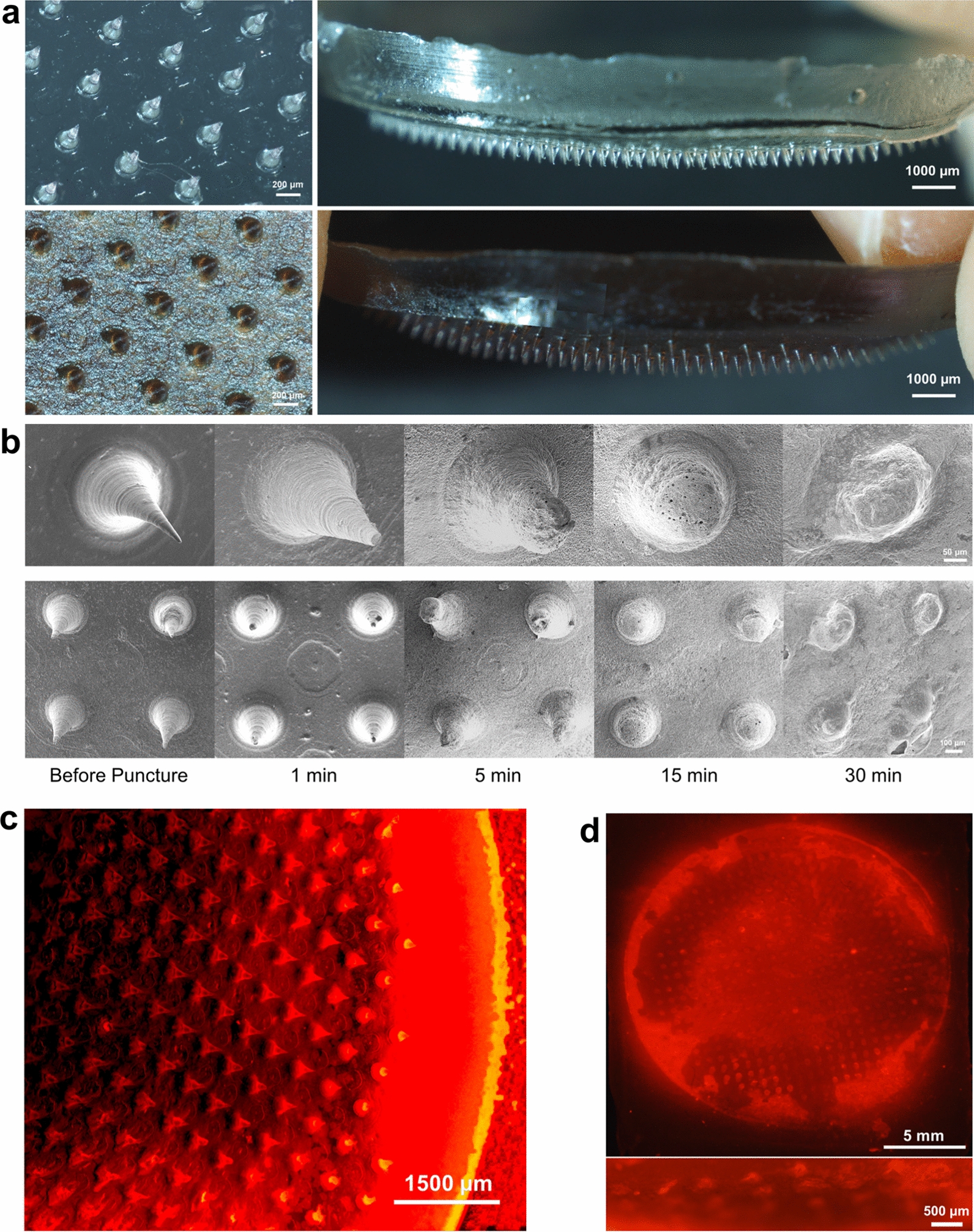
Microscopic morphologies and drug-releasing performance of the MNs patches. **a** Fluorescence stereomicroscope images of the surface morphology and overall view of drug-free (above) and drug-loaded (below) MNs. **b** SEM images of MNs before and after being pressed on mice skin for 1, 5, 15 and 30 min, respectively. **c** Fluorescence morphology image of Rhodamine B loaded MNs. **d** Fluorescence top-view (above) and section-view (below) images of mice skin treated by Rhodamine B loaded MNs for 1 min

As a widely used polymer biomaterials in the medical and food industries, sodium carboxymethylcellulose (SCMC) owns brilliant water solubility properties and excellent biocompatibility, making it be an ideal substrate material together with its sufficient mechanical strength and handy processing [29]. Through the one-step suction exhaust casting method using the polydimethylsiloxane (PDMS) molds as templates, drug-loaded and drug-free microneedles were successfully fabricated, showing conical needle tips connected by a circular base (Fig. 2a). The round base diameter of the fabricated microneedle patches was 17.5 mm. The bottom size of a single needle tip was about 270 μm and the height of each tip was about 500 μm to guarantee penetration through the dermis. Previous experiments confirmed that after the irradiation of UV or natural light, sodium nitroprusside (SNP) would release NO after reacting with further oxidized Fe^2+^. During this reaction, sedimentary Prussian blue analogues were generated in situ, and the color of mixture would gradually shift from dark brown yellow to blue, a color indicating potential photothermal capability (Additional file 1: Fig. S1a) [25]. Fourier transform infrared spectroscopy (FTIR) was also employed to characterize the mixture of SNP and ferrous lactate after irradiation by UV. As shown in Additional file 1: Fig. S1b, the FTIR spectrum revealed the corresponding characteristic peaks of C=O and –CN– at wavenumbers of 1660 and 2090 cm^−1^ after the activation of UV, which was consistent with Prussian blue analogue in previous research [30]. The X-ray diffraction (XRD) pattern of the nanoparticles mixture of SNP and ferrous lactate before and after the exposure to UV was also captured, and the peaks at 2θ = 17.4°, 24.6°, 35.2°, and 39.5° corresponding to the in mixture after the activation of UV were assigned to the (200), (220), (400), (420) planes, displaying ideal crystallinity and successful fabrication of Prussian blue analogues (Additional file 1: Fig. S1c) [30]. To confirm whether the mixed solution would generate absorption peak in NIR region, 0.1 M SNP solution and 0.1 M FeCl_2_ solution were mixed evenly and the absorption spectrums of the solution were recorded by a UV–Vis spectrophotometer before and after 10-min UV irradiation. The absorption spectrums showed obvious absorption peaks in near infrared region after UV irradiation, while no obvious absorption peaks before irradiation of UV light (Additional file 1: Fig. S1d).

To evaluate the puncture ability and tissue interaction property of the as-obtained MNs, the morphology of microneedles after pressing on skin for different times was observed by the SEM. It was found that the tips could be completely absorbed in about 30 min (min) (Fig. 2b). Furthermore, rhodamine B, which has a similar molecular weight to SNP, was utilized to simulate the percutaneous releasing process of drugs administered by MNs (Fig. 2c). The drug-loaded tips had effectively penetrated into the skin at the early stage within 1 min, which was detected from the top vertical view and cross section of the mice's skin (Fig. 2d).

Meanwhile, after pressing the MNs on mice's skin for 1 min, effective punctures and micro holes were gradually closed within 20 min, showing integration ability with surrounding tissues (Additional file 1: Fig. S2). Since SNP has the potential antihypertensive effect and accumulated harmfulness of nitrites, in order to explore the optimal drug combination for the subsequent experiments, the dosage of drug packaged in each SNP-Fe@MNs patch was designed to load gradient concentrations of SNP based mainly on the rats median lethal dose (LD_50_)) of intravenous injection 11.2 mg kg^−1^ and subcutaneous injection 9.3–13.9 mg kg^−1^ [31–33]. Thus, to achieve a balanced between therapeutic effect and drug safety, gradient concentrations of 0.01, 0.03, 0.05, 0.10, and 0.15 g mL^−1^ SNP with the molar ratio 1:1 of Fe^2+^ were loaded in SNP-Fe@MNs for further investments.

**Fig. 3:**
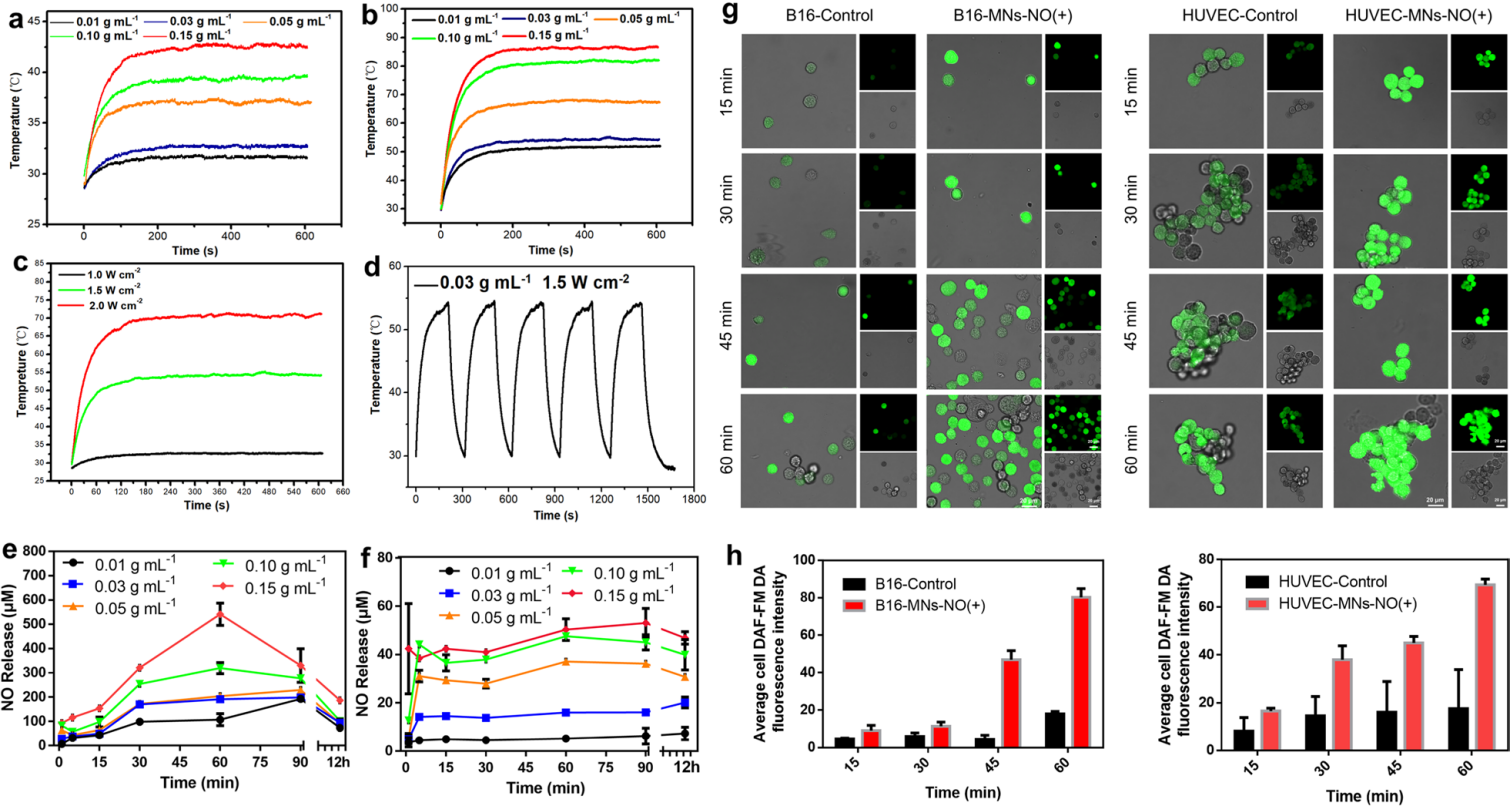
The photothermal and NO releasing performances of SNP-Fe@MNs. **a** The heating curves of SNP-Fe@MNs loading loaded with 0.01, 0.03, 0.05, 0.10 and 0.15 g mL^−1^ SNP and Fe^2+^ under NIR irradiation at 1.0 W cm^−2^. **b** The heating curves of SNP-Fe@MNs loading 0.01, 0.03, 0.05, 0.10 and 0.15 g mL^−1^ SNP and Fe^2+^ under NIR irradiation at 1.5 W cm^−2^. **c** The heating curves of SNP-Fe@MNs loaded with 0.03 g mL^−1^ SNP and Fe^2+^ under varied power densities of NIR irradiation. **d** The cyclic heating curves of SNP-Fe@MNs loaded with 0.03 g mL^−1^ SNP and Fe^2+^ under NIR irradiation at 1.5 W cm^−2^. **e** The NO releasing curves of SNP-Fe@MNs loading 0.01, 0.03, 0.05, 0.10 and 0.15 g mL^−1^ SNP and Fe^2+^ under UV irradiation. **f** The NO releasing curves of SNP-Fe@MNs loading 0.01, 0.03, 0.05, 0.10 and 0.15 g mL^−1^ SNP and Fe^2+^ without UV irradiation. **g** The intracellular NO detected with 3-Amino, 4-Aminomethyl-2′,7′-difluorescein, diacetate (DAF-FM DA) fluorescence probe in B16 and HUVECs. **h** The intracellular fluorescence intensity of DAF-FM DA probe in B16 and HUVECs

The photothermal potency of biomaterials that are applied for PTT is the indispensable yardstick that guarantees the actual therapeutic efficacy [34]. Therefore, the photothermal properties of SNP-Fe@MNs were tested by irradiating with an 808 nm NIR laser at 1.0 W cm^−2^ and 1.5 W cm^−2^. Under 1.0 W cm^−2^ NIR irradiation, the highest temperature reached around 42.4 °C at 0.15 g mL^−1^ group while the lowest was around 31.6 °C at 0.01 g mL^−1^ group (Fig. 3a). Meanwhile, under 1.0 W cm^−2^ NIR irradiation, the highest temperature reached around 86.6 °C at 0.15 g mL^−1^ group while the lowest was around 51.8 °C at 0.01 g mL^−1^ group. The temperature of 0.03 g mL^−1^ group reached around 54.2 °C within 10 min during the platform period (Fig. 3b). Though it has been reported widely that when hyperthermia reaches 43 °C would start inducing cell apoptosis of tumor, it has been proven that 52 °C is the lower threshold to guarantee thorough ablation in vivo [16]. However, implantable anti-tumor biomaterials inevitably confront the following issues that the skin barrier, position and volume of tumors, depth and size of implants, which would impact on the heat distribution and leave insufficiently heated areas [16]. However, excessive thermal ablation in situ may cause irreversible empyrosis which leads to overheating a wide range of skin epidermis, dermis, and subcutaneous tissues [35]. Above all, the temperature curves under different power densities were recorded showed attractive prospects in generation capability, which reached the threshold-temperature with the minimum concentration of 0.03 g mL^−1^ (Fig. 3c). Therefore, SNP-Fe@MNs loading 0.03 g mL^−1^ SNP and Fe^2+^ was chosen for the subsequent experiments, Besides, SNP-Fe@MNs displayed conspicuous thermal stability in cyclical heating–cooling process (Fig. 3d).

Apart from PTT, the NO gas releasing capacity was also evaluated. Since the reaction was triggered by UV light, SNP-Fe@MNs loading 0.01, 0.03, 0.05, 0.10 and 0.15 g mL^−1^ SNP and Fe^2+^ were immersed in 1 mL of PBS and treated with UV light for 1 min. The curves started to decline or stop rising at 60 min after UV irradiation (Fig. 3e). When the UV was removed, there was still a small amount of NO because it cannot be completely avoided that the light irradiation in environment will continue the process (Fig. 3f). Next, the actual amount of NO that entered into B16 and HUVECs cells was detected by DAF-FM DA fluorescence probes, respectively (Fig. 3g). The intracellular fluorescence probe intensity was then analyzed by Image J software (Fig. 3h). It was found that from 15 to 60 min the intracellular NO gradually accumulated, indicating the feasibility of utilizing NO released from SNP-Fe@MNs as gas therapy agents. Furthermore, no significant differences were observed between groups with or without 808 nm laser, demonstrating that the NIR irradiation cannot trigger NO release (Additional file 1: Fig. S1e).

**Fig. 4:**
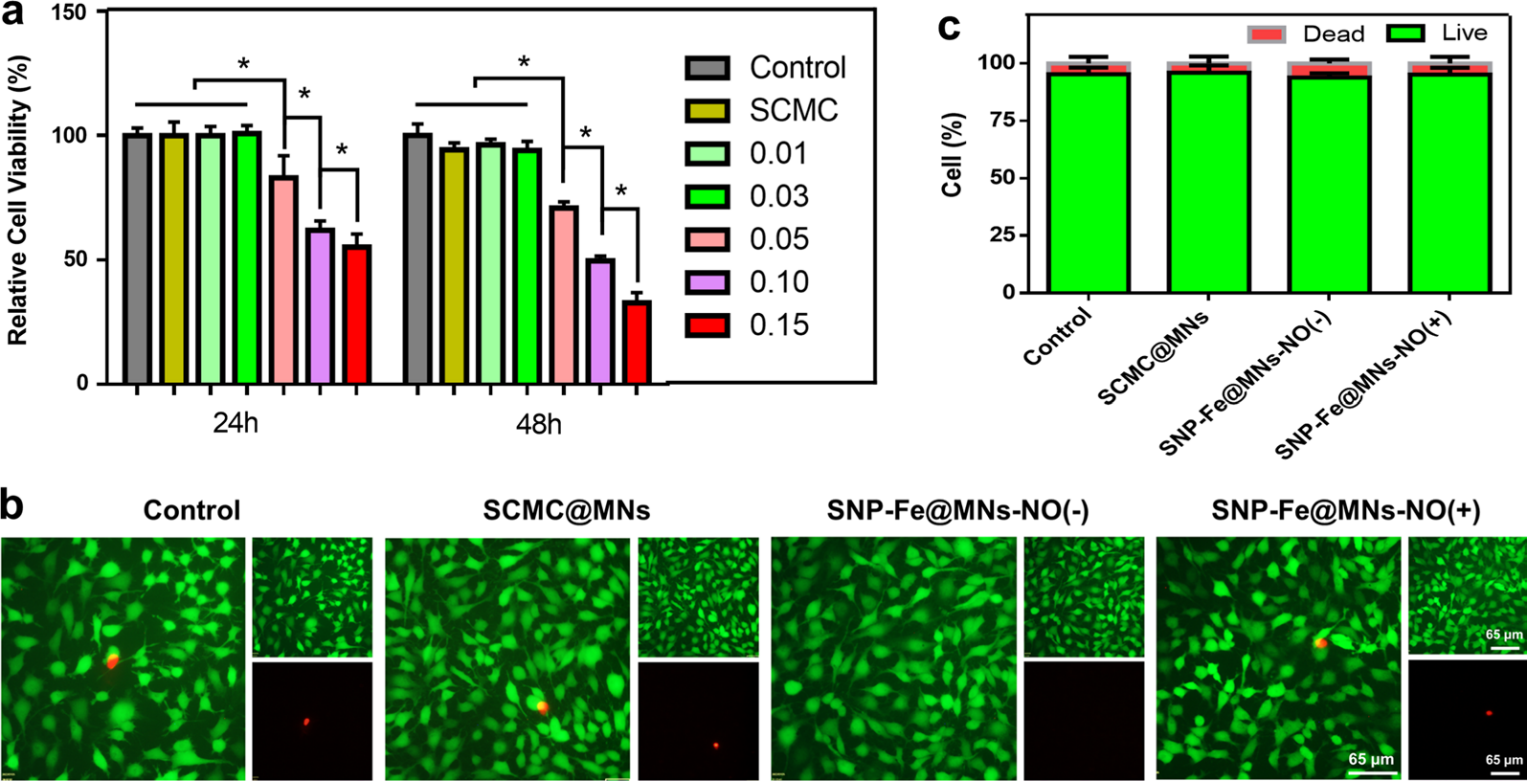
The biocompatibility of MNs. **a** Cell viability of BMSCs after culturing with SCMC@MNs, SNP-Fe@MNs loading 0.01, 0.03, 0.05, 0.10, and 0.15 g mL^−1^ SNP and Fe^2+^ for 24 h and 48 h, respectively. **b** CLSM images of HUVECs stained with the Calcein-AM/PI Kit from the Control, SCMC@MNs, SNP-Fe@MNs-NO(−), SNP-Fe@MNs-NO(+) groups. **c** Relative proportion of living and dead cells (n = 5. *P < 0.05; **P < 0.01; ***P < 0.0001)

Biosafety of new-designed therapeutic agents is crucial and fundamental to future clinical application. The biocompatibility of the MNs was investigated through in vitro cell assays. Firstly, the biosafety of SCMC@MNs, SNP-Fe@MNs loading 0.01, 0.03, 0.05, 0.10 and 0.15 g mL^−1^ SNP and Fe^2+^ was evaluated via the CCK-8 assay with no light irradiation. After co-culturing with the aforementioned MNs for 24 and 48 h (h) respectively, the relative cell viability of mouse fibroblast cells L929 showed no statistically significant difference between control and group loading 0.03 g mL^−1^ agents. Viability decreased negligibly with the gradually increasing concentration of SNP (Fig. 4a). These results suggested that from the group loading 0.05 g mL^−1^ agents onward, increasing concentration indicated ascending biotoxicity and the critical group loading 0.03 g mL^−1^ SNP and Fe^2+^ was selected for the follow-up experiment, which was consistent with the results screened in the above photothermal tests. Furthermore, the biocompatibility of SNP-Fe@MNs was confirmed via staining HUVECs with Calcein AM/PI kit and the quantitively analyzed results were arranged in the proportion diagram of living and dead cells (Fig. 4b, c). Whether MN patches were irradiated by UV or not in the 0.03 mL^−1^ group, there was no statistically significant differences among different groups, which indicated the biocompatibility of SNP-Fe@MNs reached the basic requirement for further studies and applications.

**Fig. 5:**
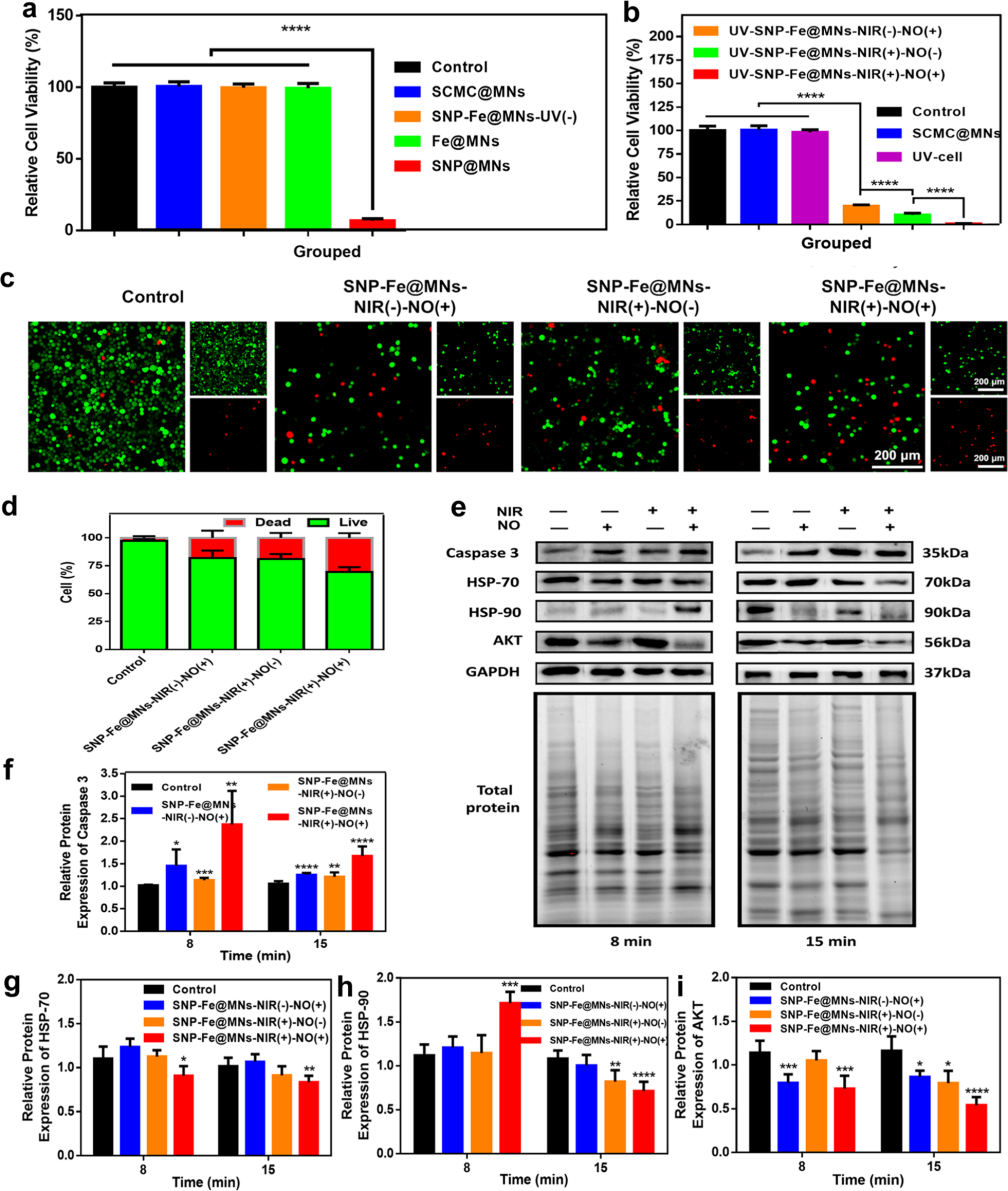
The antitumor therapeutic efficacy of SNP-Fe@MNs in vitro. **a** Relative cell viability of B16 cells from the Control, SCMC@MNs, SNP-Fe@MNs-UV(-), Fe@MNs, and SNP@MNs groups. **b** Relative cell viability of B16 cells from the Control, SCMC@MNs, UV-cell, UV-SNP-Fe@MNs-NIR(−)-NO(+), UV-SNP-Fe@MNs-NIR(+)-NO(−), and UV-SNP-Fe@MNs-NIR(+)-NO(+) groups. **c** CLSM images of B16 cells stained via the Calcein-AM/PI Kit from the Control, SNP-Fe@MNs-NIR(+)-NO(−), SNP-Fe@MNs-NIR(−)-NO(+), SNP-Fe@MNs-NIR(+)-NO(+) groups. **d** The relative proportion of living and dead cells. **e**–**i** Protein levels of Caspase-3 (**f**), HSP-70 (**g**), HSP-90 (**h**), and AKT (**i**) after treated by MNs (n = 6. *P < 0.05; **P < 0.01; ***P < 0.0001)

The involvement of divalent iron Fe^2+^ may induce iron-dependent ferroptosis-mediated programmed cell death [36]. To confirm whether ferroptosis was involved in tumor ablation, B16 cells were cocultured with SCMC@MNs, SNP-Fe@MNs-UV(−), Fe@MNs, and SNP@MNs, separately. The CCK-8 assay showed that Fe@MNs made no difference with the former three groups. However, SNP@MNs displayed a significant decline in cell viability, which might due to the accumulation of [Fe(CN)_5_]^2−^ while SNP was gradually decomposed, indicating that loaded Fe^2+^ probably would rather rescue the toxicity of reaction-product of SNP than induce ferroptosis. Nevertheless, when SNP is compatible with Fe^2+^, the side effects of the drug were reversed (Fig. 5a). Subsequently, the tumor-ablation performance was tested in vitro by co-culturing B16 cells with SNP-Fe@MNs. The CCK-8 assay showed the relative cell viability of group UV-cell had no significant difference with that of group Control, inferring that the process of UV irradiation lasting for 1 min had no statistical impact on cells. Moreover, the cell viabilities in groups UV-SNP-Fe@MNs-NIR(−)-NO(+), UV-SNP-Fe@MNs-NIR(+)-NO(−) and UV-SNP-Fe@MNs-NIR(+)-NO(+) was hierarchically suppressed, indicating that the combination of PTT and gas therapy obtained completely ablation of tumor cells (Fig. 5b). Furthermore, after coculturing for 24 h, the antitumor property of MNs was confirmed via Calcein AM/PI staining and the overall trend was consistent with CCK-8 assay, indicating a huge potential of SNP-Fe@MNs for the thorough ablation of melanoma (Fig. 5c, d). Meanwhile, the migration ability of B16 after treating with SNP-Fe@MNs was evaluated by scratch assay and the number of migrated cells to middle of scratch in group SNP-Fe@MNs-NIR(+)-NO(+) was the least among all groups, indicating favorable restriction of tumor invasiveness and prevention of tumor recurrence (Additional file 1: Fig. S3).

Moreover, B16 was cocultured with MNs for 24 h and then treated with PTT for 8 min and 15 min individually at the next day. To clarify ablation-related mechanisms and pathways that high concentration of NO gas accumulated around the SNP-Fe@MNs and diffused into cells along the concentration gradient, reaching toxic levels that can be exploited in achieving direct apoptosis of tumor cells through heme nitrosylation of cytochrome-c, which may enhance its proapoptotic function by promoting caspase-3 activation, the expression of apoptosis marker was detected by Western blot (Fig. 5e). The expression of caspase-3 was analyzed, showing up-regulated level in groups SNP-Fe@MNs-NIR(−)-NO(+), SNP-Fe@MNs-NIR(+)-NO(−), and SNP-Fe@MNs-NIR(+)-NO(+). Groups SNP-Fe@MNs-NIR(+)-NO(+) exhibited the highest expression of caspase 3 among these groups, indicated that at both 8-min and 15-min stage, cell apoptosis was more inclined to the impact of synergetic NO burst release and hyperthermia (Fig. 5f). However, we then found that the expression level of caspase-3 at 8-min in group SNP-Fe@MNs-NIR(+)-NO(+) was rather unstable, because there was a rather large SD value when we repeated the WB analysis for three times, which meant that the reactions of B16 cells towards the collaborative therapy was irregular or unpredictable at the 8-min early stage. Thus, we surmised that in the early stages some cells may experience a sharp increase in compensatory response to stimuli. But in the later stages, their response may be more stable and displayed as a convergence state.

Researches focused on the role of HSPs on malignant cells suggested that tumor cells are “addicted to chaperones” [37]. On account of surviving under continuous nutrient deficiency, hypoxia, and other stress conditions, tumor cells are prone to apoptosis [38]. HSP-70, abundant in cancers, was proved to inhibit apoptosis through caspase-dependent mechanisms by suppressing c-Jun N-terminal kinase (JNK), caspase-independent mechanism by suppressing apoptosis-inducing factor (AIF) and via interactions with death receptors. As a consequence, a decline in the level of HSP-70 was proved to induce cancer cell death [39]. To further identify the signaling pathways that were involved in the induction of caspase-3-dependent apoptosis in B16 cells treated with MNs, the expression of several cell survival related protein HSP-70, HSP-90, and AKT was studied in follow.

The expression level of HSP-70 in group NIR(+)-NO(+) decreased significantly, suggesting that synergistic therapy can overcome the apoptosis protection mechanism possibly caused by the HSP-70 (Fig. 5g). As a heat stress marker, the expression of HSP-90 showed an early surge as the resistance to damage caused by gradually increased heat stress and NO gas [40]. Since HSP-90 was related to the thermo-tolerance protective mechanism towards heat stress. It was worth noting that single NO gas or hyperthermia therapy cannot provoke the cellular self-protection mechanism of HSP-90. The higher level indicated the protective mechanism confronting the combination treatment of NO and hyperthermia at 8-min. However, the situation had been reversed over time at 15-min with the down-regulation of HSP-90 levels in the PTT group and the synergistic therapy group. With the decrease of thermo-tolerance protective HSP-90, which was mainly aimed at confronting heat stress, cells began to undergo apoptosis over time (Fig. 5h). As another cell survival marker, the expression of AKT was significantly decreased in the NO-gas group and the synergistic-therapy group after 8-min-treatment, demonstrating the obvious effect of shock therapy of NO at early stage. However, this survival index decreased after 15 min of treatment with the utmost variable significance in the synergistic therapy group (Fig. 5i). Thus, the apoptosis of malignant cells might be induced by the up-regulation of caspase-3, and came up with the decline in HSP70, HSP-90, and its client protein, AKT, also known as HSP-90/AKT signaling pathways (Additional file 1: Fig. S4).

**Fig. 6:**
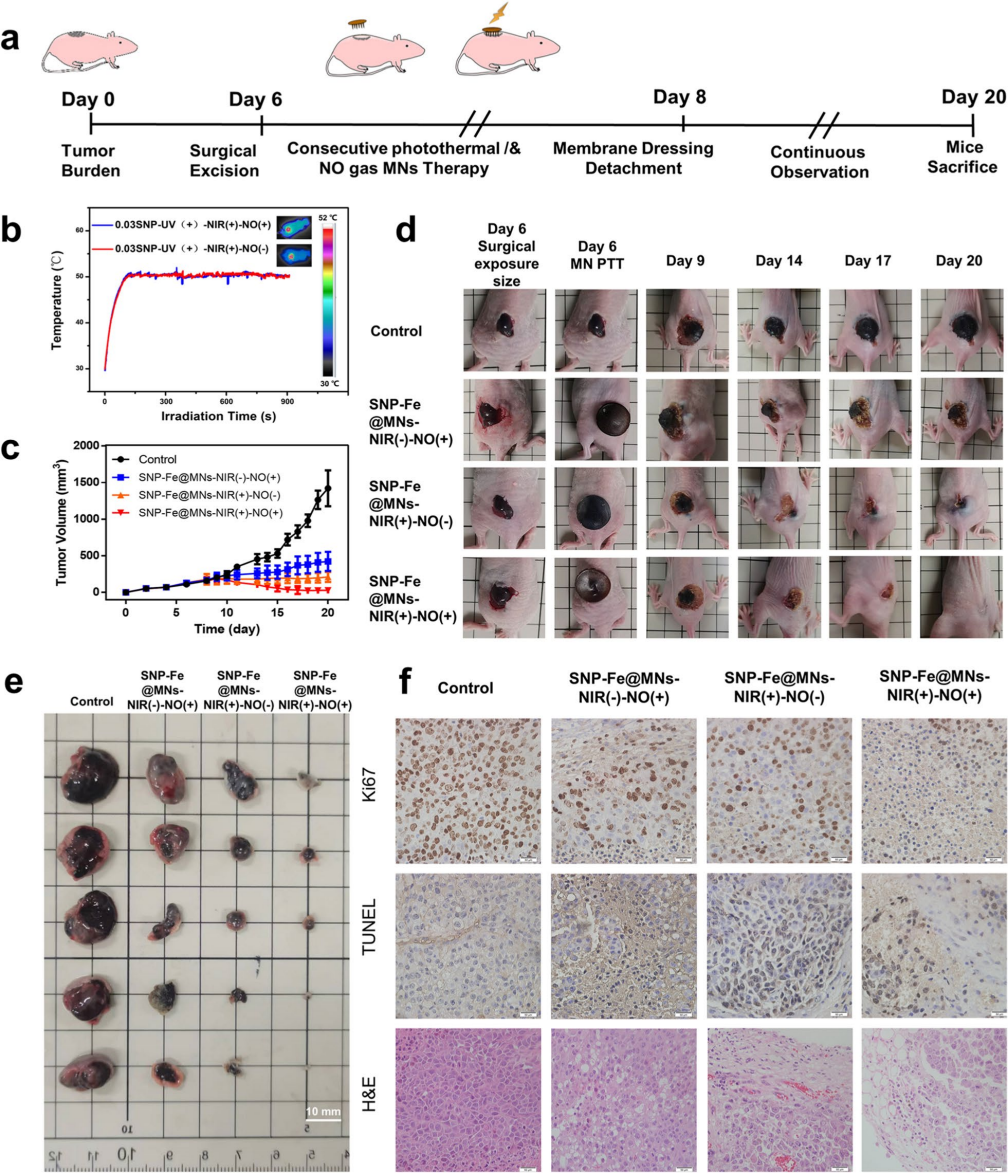
The in vivo antitumor therapeutic efficacy of SNP-Fe@MNs. **a** Conceptual design of postoperative B16 tumor cell residues with defect model. **b** In vivo heating curves and infrared thermal images of mice under the irradiation of 1.50 W cm^−2^ NIR. **c** Tumor volume in groups of control, SNP-Fe@MNs-NIR(−)-NO(+), SNP-Fe@MNs-NIR(+)-NO(−), and SNP-Fe@MNs-NIR(+)-NO(+), respectively. **d** Photographs of the whole therapeutic course. **e** Photographs of dissected tumor. **f** Ki67 immunohistochemical, TUNEL, and H&E staining images of a dissected tumor tissues in different treatment groups

Inspired by the in vitro effect of MNs from the aforementioned experiments, malignant melanoma B16-bearing nude mice were utilized for further investigation on the in vivo antitumor performance of MNs. On day 6, when the volume of the tumor reached around 100 mm^3^, full-thickness wounds slightly wider to adhere to the tumor body were surgically produced to simulate the recurrence of tumor after resection. After surgical exposure, the wound was then penetrated with different types of MNs and fixed with a transparent membrane dressing on the surface during the therapy (Fig. 6a). For the NIR(+) group, a 808 nm laser (1.50 W cm^−2^, 15 min) was applied after the utilization of the MN patches from days 6 to 8. During the consecutive treatments, the ranges of temperature in the wound area were recorded by the infrared camera. The temperature reached about 52 °C and stayed in a platform stage, whether in the NIR(+) group or in the NIR(+)-NO(+) group, which ensured the basic requirement of reasonable heat distribution to induce apoptosis and prevent unnecessary burns to surrounding tissues (Fig. 6b). From day 8, when the microneedles covered on postsurgical area were almost absorbed and dissolved, the tumor volume, images, and body weight of mice were recorded (Fig. 6c, d and Additional file 1: Fig. S5). From day 9, the curative effect has changed intuitively at the NO group, PTT group, and cooperative NO-PTT group, the size of the tumor presented a stepwise decline. However, the tumor volume in the control group showed an uncontrollable, similar exponential increase. On day 20, the original enlarged wound of the NIR(+)-NO(+) group had been completely closed with no intuitive tumors left. Subsequently, the tumors of each group were dissected and recorded by digital photograph (Fig. 6e). Then, the weight of each tumor was recorded and then soaked in paraformaldehyde for further study (Additional file 1: Fig. S6). As a cell proliferation marker, Ki67 expression was detected with immunofluorescence staining, showing the lowest expression in the NIR(+)-NO(+) group, and the highest expression in the Control group. In cyanine-3 dye-conjugated TdT-mediated dUTP nick end labeling kit (TUNEL) staining assays, the highest rate of positive cells stained to be brown was the NIR(+)-NO(+) group, which indicated the maximal proportion of apoptotic tumor cells. The H&E staining displayed cell shrinkage, vacuolation, and the absence of nuclei to varying degrees among different groups after treatment. Remarkably, the smallest tumor body and scarcest tumor cells were found in the synergistic gas-hyperthermia therapy group (Fig. 6f).

Although it can be found intuitively and confirmed statistically that PTT is superior to gas therapy in inhibiting tumor progression, however, a single strategy will inevitably lead to tumor recurrence to a certain extent after long-term observation. Comparatively, the synergistic gas-hyperthermia therapy had the best therapeutic effect in vivo*,* which was consistent with the former conclusions, suggesting favorable potential for clinically application. Simultaneously, biosafety and biocompatibility were further confirmed via the bodyweight diversity curves and H&E staining of the main organs (skin, heart, lung, liver, spleen, and kidney), indicating that no obvious pathological abnormality was observed (Additional file 1: Figs. S5, S7).

**Fig. 7:**
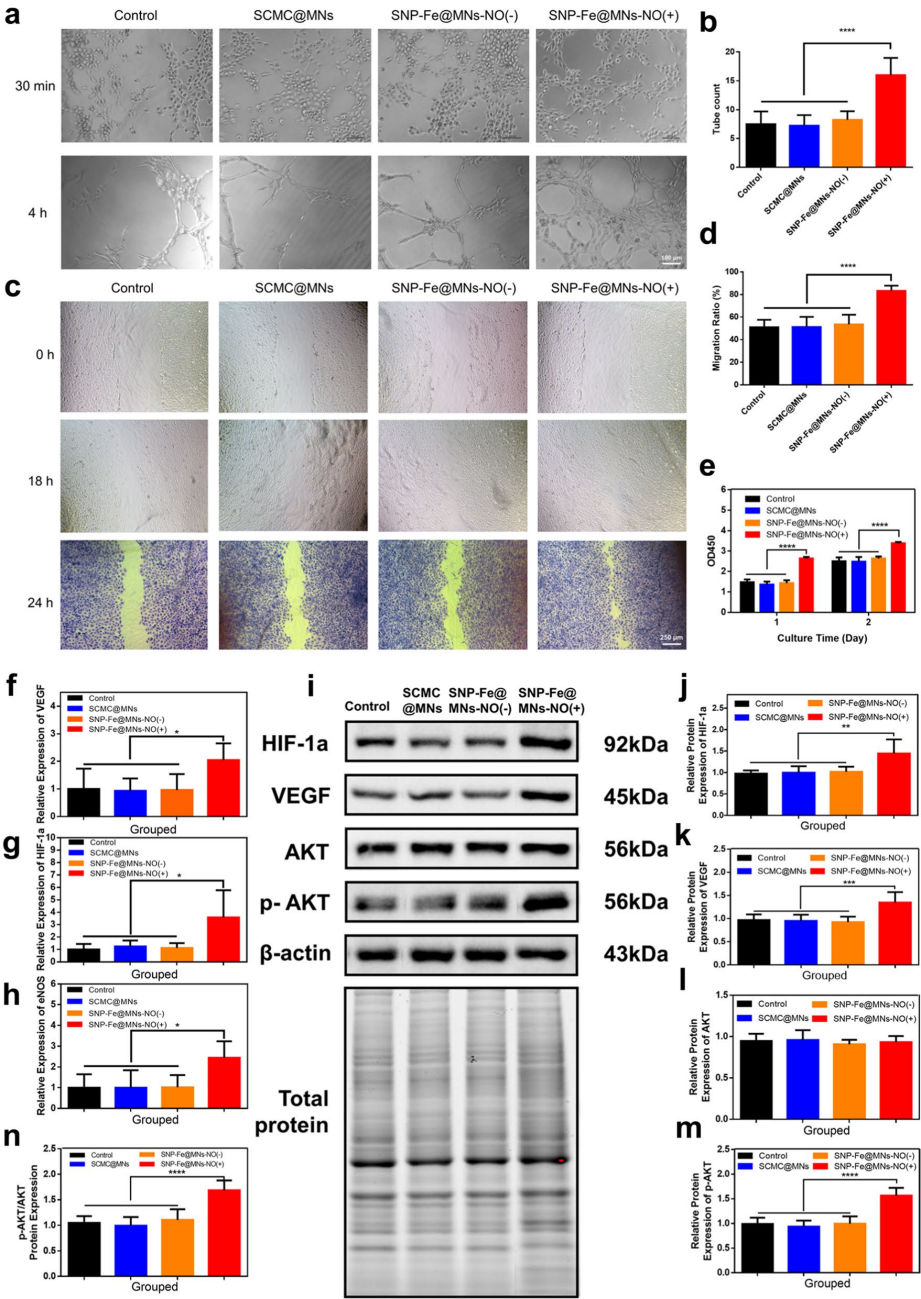
The in vitro bioactive effect of the NO gas on the proliferation, adhesion and angiogenic performance of HUVECs. **a** Images of the tube formation experiment with HUVECs in the control, SCMC@MNs, SNP-Fe@MNs-NO(−),and SNP-Fe@MNs-NO(+) groups. **b** The relative data is expressed as the mean ± S.D. from three independent experiments. **c**, **d** Scratch images of HUVECs migration after coculture with SCMC@MNs, SNP-Fe@MNs-NO(−), and SNP-Fe@MNs-NO(+) groups and relative migration ratio. **e** OD 450 values of HUVECs after being treated by MNs for 1 and 2 days. **f**–**h** Angiogenesis-related gene expression of VEGF, HIF-1a, and eNOs in HUVECs. **i**–**n** Protein levels of HIF-1a, VEGF, AKT, p-AKT, and ratio of p-AKT/AKT after treated by MNs were detected by Western blot (n = 6. *P < 0.05; **P < 0.01; ***P < 0.0001)

The bioactivity of MNs in vitro was subsequently proven by the tube formation experiment. The gradual release of NO in the SNP-Fe@MNs-NO(+) group enhanced the proliferation, migration and tube formation capacity of HUVEC than the other groups (Fig. 7a). The quantities of the tubes were 7.500 ± 0.7792, 7.250 ± 0.6478, 8.250 ± 0.5261, and 16.00 ± 1.052 in the Control, SCMC@MNs, SNP-Fe@MNs-NO(−), and SNP-Fe@MNs-NO(+) groups, respectively (Fig. 7b).

Meanwhile, the migration ability of HUVEC incubated with the 1640 extracts of different types of MNs was displayed via scratch assay in order to imitate the process of in vitro wound healing. The width of around 550 µm vertical scratches was formed by a 200 µL pipette tip. The migrations of HUVECs were observed after 18 h and 24 h via a microscope and analyzed by Image J (Fig. 7c, d). The migration ratio was (83.34 ± 2.961)% in the SNP-Fe@MNs-NO(+) group in 24 h compared with that of around 51% in the other three groups, strongly indicating that the sustained release of NO plays a prominent role in promoting endothelial cell migration. Meanwhile, based on the results of the CCK-8 assay, the proliferation of HUVECs incubated with SNP-Fe@MNs-NO(+) was significantly enhanced than that of cells treated with SCMC@MNs and SNP-Fe@MNs-NO(−) (Fig. 7e).

Previous researches had demonstrated that the mechanisms by which NO stimulates angiogenesis were not fully clarified. NO, as an endothelial survival factor, inhibits apoptosis and enhances endothelial cell proliferation by improving the expression of VEGF [41, 42]. To make further investigation about how NO exerts its angiogenic effects, RT-PCR analysis of HUVEC cells after coculturing for 5 days with SCMC@MNs, SNP-Fe@MNs-NO(−), and SNP-Fe@MNs-NO(+) was performed. Results showed that NO could upregulate the expression of angiogenic genes, including VEGF HIF-1α, and eNOs, compared with the other three groups without NO, suggesting NO released by MNs had the ability to promote angiogenesis by upregulating expression of related genes in vitro (Fig. 7f–h).

Meanwhile, under the same condition, the angiogenesis-related mechanism mechanisms and pathways were studied by Western Blot with a sample of HUVEC cell lysates (Fig. 7i). The results showed that the expression levels of HIF-1a and VEGF were significantly enhanced in groups with the existence of NO (Fig. 7j, k). Regarded as another fatal survival index, the expression levels of AKT showed no statistical difference among the four groups (Fig. 7l), however, the level of p-AKT ascended in groups with NO in contrast (Fig. 7m), which indicated that the existence of NO activated the phosphorylation of AKT (Fig. 7n). It is reasonable to speculate that SNP-Fe@MNs-NO(+) as a NO-donor can regulate the expression of HIF-1a and VEGF via the phosphoinositide 3-kinase (PI3K)/AKT signaling pathways, in addition to promoting secretion of VEGF by vascular smooth muscle cells [42, 43].

**Fig. 8:**
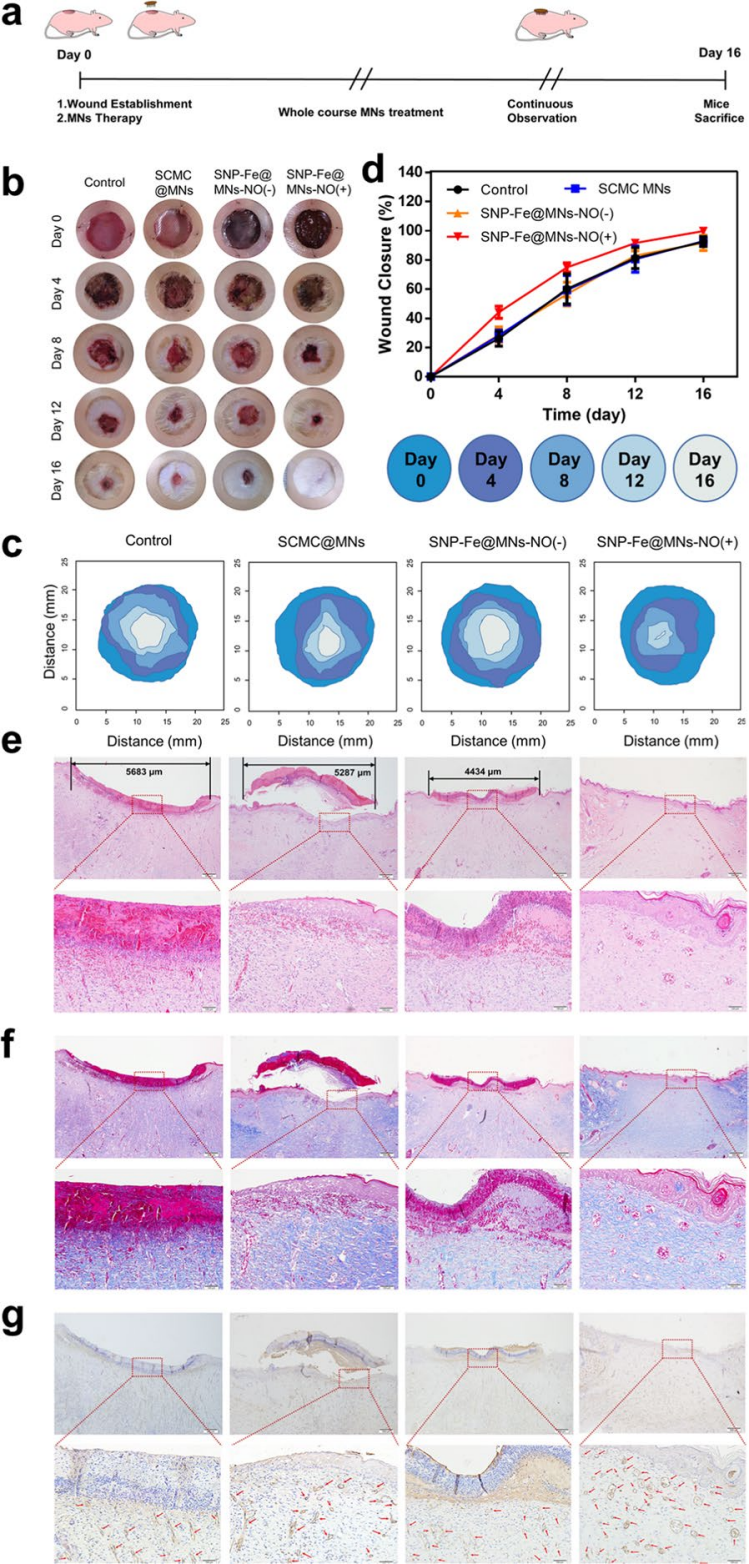
The tissue regeneration performance of SNP-Fe@MNs in vivo. **a** Conceptual design of a postoperative full-thickness defect model. **b** Photographs and the related (**c**) the simulated variation images of wound morphology and size at five time points. **d** Statistical curve of wound closure rate among different groups. **e** H&E, **f** Masson, and **g** CD31 immunohistochemistry staining images of the dissected wound tissue among four groups after 16 days

The ability to promote tissue regeneration of maxillofacial skin is essential for early healing to reduce infection risks, enhance recovering speed and avoid protracted course in patients with post-operative wounds [44]. Encouraged by the considerable results of in vitro bioactivity experiments, a full-thickness cutaneous defect model was utilized on the left flank of the SD rats to mimic the large open injuries after routine extended surgical excision of melanoma. The diameter of the in vivo round skin coloboma was prepared to be 17 mm via a skin drill, slightly less than 17.5-mm-diameter MN patch. Then the tailored silicone rings were sewn to fix around the wounds accordingly to prevent shrinking. After the wound had been established, MNs were applied to the wound of the corresponding group for the whole course from day 0 to day 16 (Fig. 8a). Considering the long-term metabolism and degradation of the biomaterial, in order to ensure the overall efficacy, dressings were changed for the wounds every 4 days, which synchronized with the photographic frequency of the healing process to track the change in wound size (Fig. 8b). Apart from intuitive photographs, simulated wound size and morphology images displayed the effect of SNP-Fe@MNs-NO(+) in promoting wound closure and healing (Fig. 8c). In addition, MNs with NO had exceptional ability to promote wound healing, especially in the early stage, showing that the wound closure rate was much higher than other groups, which would help reducing the incidence of adverse effects. On the 16th day, the wound closure rate reached almost 100% in the group with NO gas (Fig. 8d). By the end of day 16, the wound beds of full-thickness recovery were dissected and analyzed by H&E staining, Masson staining and CD31 immunohistochemical staining. The results of H&E staining showed that apparent scars and beneath immature granulation tissue had lengths of 5683 μm, 5287 μm and 4434 μm in the control, SCMC@MNs, and SNP-Fe@MNs-NO(−) groups, respectively. The SNP-Fe@MNs-NO(+) group displayed the ignorable immature granulation tissue and fully recovery with new functional epidermis and dermal tissue, distinct demarcation, and skin appendages such as hair follicles, glands, and capillaries rather than cicatricial tissue healing (Fig. 8e). Consistent with the results of H&E staining, the Masson staining showed the blue and dense revitalized collagen fiber layer in groups with NO (Fig. 8f). It is widely known that revascularization in the wound beds is vital for skin regeneration, and thus, the expression of CD31 was analyzed via immunohistochemical staining [45]. Notably, the maximum number of CD31 positive vascular vessels stained to be brown were found in groups with NO, which was marked with red arrows, confirming the indispensable role of NO in neovascularization in vivo (Fig. 8g).

## Conclusion

In summary, a novel multifunctional SNP-Fe@MNs patch was fabricated as a controllable hyperthermia and sustaining gas-therapy platform for the ablation of maxillofacial malignant skin tumors and associated tissue regeneration. As a transdermal drug delivery system, MNs could penetrate through the dermis to exhibit appreciable photothermal capabilities and realize the burst release of NO to induce the apoptosis of tumor cells via caspase-3-related HSP-70/HSP-90/AKT signaling pathways. Furthermore, to accomplish the promising postoperative tissue regeneration, the long term steadily releasing of low concentration NO gas by the SNP-Fe@MNs in vivo and in vitro could guarantee the angiogenic effect via upregulating HIF-1a/VEGF through the PI3K/AKT signaling pathway. Considering the therapeutic dilemma of skin malignancies, the multifunctional SNP-Fe@MNs route could conquer the postoperative residual and recurrence melanoma tumor cells and stimulate the surrounding skin tissue in one step, suggesting that the bio-application of SNP-Fe@MNs could be further popularized as an remedy for synergistic therapy in the treatment of maxillofacial melanoma.

## Experimental section

### Materials and reagents

Na_2_[Fe(CN)_5_NO]·2H_2_O and [CH_3_CH(OH)COO]_2_Fe·xH_2_O were purchased from Sigma-Aldrich, USA. Carboxymethyl cellulose (SCMC; MW. ~ 90,000, #C104983), crystal violet, and rhodamine Bwere purchased from Aladdin, China. Phosphate buffer solution (PBS), minimum essential medium (MEM), Roswell Park Memorial Institute (RPMI) 1640 medium, trypsin, penicillin, streptomycin, and fetal bovine serum (FBS) were purchased from Gibco, USA. Cell Counting Kit-8 (CCK-8), antibody dilution buffer, radioimmunoprecipitation assay (RIPA) lysate, Total Nitric Oxide Assay Kit, and DAF-FM DA were purchased from Beyotime, China. A cyanine-3 dye-conjugated TdT-mediated dUTP nick end labeling kit (TUNEL), distilled RNase-free water, a bicinchoninic acid (BCA) protein assay kit, TRIzol RNA isolation reagents, RevertAid first strand cDNA synthesis kit and polyvinylidene difluoride membranes were purchased from Thermo Scientific, USA. 2X SYBR Green Pro Taq HS Premix qPCR kit was purchased from Accurate Biology, China. Calcein AM/PI Double Staining Kit, paraformaldehyde (PFA, 4%), and hematoxylin–eosin (H&E) Staining Kit were purchased from Solarbio, China. One light-curing resin component was purchased from Friedrichsdorf, Germany. A PDMS microneedle mold and negative pressure centrifugal pump were purchased from Engineering for Life, China. The SDS-PAGE kit was purchased from Bio-Rad, USA. Polyvinylidenedifluoride membrane (PVDF) was purchased from Millipore, Bedford, MA, USA. NcmBlot Rapid Transfer Buffer, NcmBlot Blocking Buffer, and Ncm Enhanced Chemiluminescent (ECL) High were purchased from NCM Biotech, China. The primary antibodies used were: hypoxia inducible factor-1 (HIF-1a), vascular endothelial growth factor (VEGF), AKT, p-AKT, heat shock protein-70 (HSP-70), heat shock protein-90 (HSP-90), Caspase-3, β-actin and glyceraldehyde-3-phosphate dehydrogenase (GAPDH), all of which were purchased from Immunoway USA. The secondary antibodies were purchased from Immunoway in the USA. Other antibodies, Ki67 and CD31, were purchased from Abcam, USA. Matrigel was purchased from ABW, China. All reagents were used without further purification.

### Preparation of SNP nanoparticles and fabrication of SNP-Fe@MN patches

SNP nanoparticles were firstly synthesized by dissolving in deionized water and ethanol totally, and then the dispersion of the particles was proceeded by ultrasonic vibration. After being kept at 80 °C for 24 h, the freeze-dried nanoparticles were obtained by lyophilization (Creatrust, China). SNP-Fe@MN patches were fabricated by a one-step negative pressure centrifugation casting method using polydimethylsiloxane (PDMS) molds (Engineering For Life, China). During the suction exhaust process, 250 μL 8% w/v sodium carboxymethylcellulose (SCMC) solution with a certain concentration of drugs was added into PDMS molds, centrifugated at − 0.08 MPa in the suction pump (Engineering For Life, China) for 3 min, then excess bubbles and solutions were discharged. The above steps were repeated 3 times, followed by 24 h of ambient drying at room temperature. Drug gradient concentration for MNs used in this study were 0.01, 0.03, 0.05, 0.10 and 0.15 g mL^−1^ sodium nitroprusside (SNP) for each type of SNP-Fe@MNs respectively, mixture of SNP nanoparticles and [CH3CH(OH)COO]_2_Fe∙xH_2_O powders were under the molar ratio of 1:1 in deionized water, and non-drug for SCMC@MNs. Subsequently, MNs were obtained after discreetly demolding. All fabrication and storage processes were performed in dark or low light and dry conditions to avoid the degradation of MNs. The diameter of the MN mold was 17.5 mm in the bottom base with a 2-mm fillister. The microneedles were arrayed in a whole circularly arranged sequence, with a total number of 385.

### Characterization of SNP-Fe@MNs Patches

Firstly, fluorescence stereomicroscopy was performed to characterize the surface morphology and overall view of drug-loaded and drug-free MNs, respectively (AxioZoom.V16, Zeiss, Germany). Next, in order to evaluate the puncture ability of MNs towards tissues and the melting and absorption rate of MNs, scanning electron microscopy (SEM) was conducted to determine the MNs surface topography before and after the MNs were penetrated into ex vivo mouse skin in 1, 5, 15, and 30 min (Flexsem 1000, HITACHI, Japan). Meanwhile, observations on the puncture ability of the MNs after pressing for 1 min in vivo were implemented at 0, 2, 5, 10, 15, and 20 min. Then, for the sake of simulating of transdermal drug release process, rhodamine B (MW. = 479.01) was used via fluorescent dye to simulate SNP (MW. = 297.95) due to their rather similar molecular weights. Rhodamine B-loaded MNs (RB-MNs) were prepared by the same casting step as previous with only 0.03 g mL^−1^ Rhodamine B-loaded and SCMC solution to form MNs. The morphology of R-MNs patch was characterized by fluorescence stereomicroscopy (AxioZoom.V16, Zeiss, Germany). After that, RB-MN’s patches were pressed on mouse skin for 1 min, the overall top vertical view of the skin treated by R-MNs was captured, and the treated skin was sliced along the row of holes for cross sections that were captured by a fluorescence stereomicroscope to show the radial diffusion of rhodamine B at every hole and reveal the penetrability generated by MN tips. In addition, after mixing the nanoparticles of equal molar concentrations of SNP and ferrous lactate, one group was activated by ultraviolet light, and the other group was stored in the dark. After freeze-drying (Creatrust, China), the two groups of mixed nanoparticles were obtained, and the corresponding characteristic peaks were detected by FTIR (Thermo Scientific Nicolet iS50, USA) and XRD (Bruker D8 ADVANCE, Germany), respectively to ascertain the successful preparation of Prussian blue analogues. Additionally, 0.1 M SNP solution and 0.1 M FeCl_2_ solution were mixed evenly in a 1.5 mL EP tube at a ratio of 1:1. The obtained solution was vibrated by ultrasonic for 30 min, and the absorption spectrums of the solution were recorded by a UV–Vis spectrophotometer before and after 10-min UV irradiation.

### The photothermal properties and NO releasing behavior of SNP-Fe@MNs

SNP-Fe@MNs loading with 0.01, 0.03, 0.05, 0.10 and 0.15 g mL^−1^ SNP were irradiated by ultraviolet (UV) light for 1 min then put in 48-well plates. Next, all MNs were irradiated by an 808 nm NIR laser (808-5W, BOT, China) at a power density of 1.0 W cm^−2^ and 1.5 W cm^−2^ separately for 10 min. Furthermore, 0.03 mL^−1^ SNP-Fe@MNs were put in a 48-well plate irradiated by an 808 nm NIR laser at various power densities of 1.0, 1.5 and 2.0 W cm^−2^ for 10 min. Then, the 0.03 mL^−1^ SNP-Fe@MNs were cyclically heated—cooled at 1.5 W cm^−2^ by regularly turning the laser device on/off. All the photothermal evaluation experiments in the study were conducted under the monitor of an infrared camera (628c, Fortric, China).

To measure actual rate of NO releasing, SNP-Fe@MNs loading at 0.01, 0.03, 0.05, 0.10, and 0.15 g mL^−1^ SNP were immersed in 1 mL of PBS with or without 1 min of irradiation by UV light. The solutions were then extracted to 96-well plates, and levels of NO at 1, 5, 15, 30, 60, 90 min and 12 h were measured by the Griess reaction using the microplate reader at 540 nm (Biotek, USA). To determine the influences of NIR on the releasing rate of NO, the obtained SNP-Fe@MNs loading 0.03 g mL^−1^ SNP and Fe^2+^ in our work were immerged in 1 mL PBS and separated into two groups named as NIR(−) and NIR(+) respectively. Group NIR(+) were irradiated with NIR (808 nm) for 1 min. Then, the NO concentrations two groups were detected with Griess Reagent (Beyotime, China) at 1-, 15-, 30-, 60-, 90-min and 12 h under dark environment. To assess the intracellular NO level in B16 and HUVEC, cells were washed three times with PBS. DAF-FM DA fluorescence probes were added and incubated for 45 min at 37 °C. The fluorescence at excitation and emission wave lengths of 495 nm and 520 nm was then measured, accordingly. Finally, each fluorescence intensity was analyzed by image J software.

### In vitro evaluation on the antitumor performance of SNP-Fe@MNs

To verify the biosafety of the MN materials, murine fibroblast (L929, 2 × 10^4^) (Procell, China) cells were cultured in 24-well plates and cocultured on SNP-Fe@MNs loading 0.01, 0.03, 0.05, 0.10 and 0.15 g mL^−1^ SNP and SCMC@MNs for 24 h and 48 h in dim, respectively, then were washed three times by PBS. Next, 600 mL MEM with 60 mL of cell counting kit-8 (CCK-8) was added to each well then cultured in the cell incubator for 2 h. Finally, 100 mL of the solution was transferred to a 96-well plate and read at OD490 by the microplate reader and measured by the following relative cell viability equation:

$$\text{Relative Cell Viability }\left({\%}\right)=\frac{100\times \left(\text{Sample }{{\text{OD}}}_{450{\text{nm}}}-\text{Negative control }{{\text{OD}}}_{450{\text{nm}}}\right)}{\text{Untreated control }{{\text{OD}}}_{450{\text{nm}}}-\text{Negative control }{{\text{OD}}}_{450{\text{nm}}}}.$$

For further biocompatibility confirmation, human umbilical vein endothelial cells (HUVECs, 2 × 10^4^) (Procell, China) were seeded for 24 h with SCMC@MNs, SNP-Fe@MNs-NO(+), and SNP-Fe@MNs-NO(−), with the latter one pre-freeze-dried NO after UV irradiation during production. Besides, group of SNP-Fe@MNs-NO(+) was set to simulate the process of releasing NO in the later stage of wound restoration. MNs were applied 60 min after UV irradiation according to the previous gas releasing profile to get rid of burst release of NO. Then each group was stained with the Calcein-AM/PI Kit and measured by image J.

To explore whether ferroptosis was involved in the therapeutic process, Murine melanoma B16 cell line (2 × 10^4^) (Procell, China) were incubated for 24 h with SCMC@MNs, 0.03 g mL^−1^ SNP-Fe@MNs-UV(−) and the individuality of the same amount as former 0.03 g mL^−1^ SNP-Fe@MNs-UV(−), which were labelled as Fe@MNs together with SNP@MNs. Then the detection of relative cell viability by CCK-8 were conducted.

Furthermore, B16 (2 × 10^4^) were seeded in 24-well plates individually and cocultured for 24 h in groups that were divided as follows: control (processless), SCMC@MNs, UV-cell (simulate UV irradiation for 1 min when no material existed), UV-SNP-Fe@MNs-NIR(−)-NO(+), UV-SNP-Fe@MNs-NIR(+)-NO(−), and UV-SNP-Fe@MNs-NIR(+)-NO(+). SNP-Fe@MNs indicated for the 0.03 g mL^−1^ group, as the following represents the same meaning. Specifically, groups of NO(−) were pre-freeze-drying to remove NO, moreover, groups of NIR(+) were exposed to an 808 nm NIR laser at 1.5 W cm^−2^ while being monitored by the infrared camera until the temperature reached around 47 °C and was held for 15 min by reducing the output power of the laser device gradually. Then, all of the groups were cultured in the cell incubator (37 °C, 5% CO_2_) (Thermo Scientific, USA) for another 30 min in order for final CCK-8 detection. Next, B16 (2 × 10^4^) were cocultured for 24 h with SNP-Fe@MNs-NIR(−)-NO(+), SNP-Fe@MNs-NIR(+)-NO(−) and SNP-Fe@MNs-NIR(+)-NO(+), then were stained with the Calcein-AM/PI Kit and counted by image J. Moreover, the B16 migration condition using medium extracts of different MNs was presented by scratch testing and stained with crystal violet at day 2.

Then, Western blot analyses were conducted with the same group and the same condition as stated above, except for adjusting the photothermal time to 8 min and 15 min so as to explore the optimal time and related mechanisms. After extracting total proteins with RIPA buffer, the concentration was determined by the BCA protein assay kit. Samples each with 15 μg of protein were separated in SDS-PAGE gels, as each group was loaded together with representative front lysates from the Control group for standardization, then rapidly transferred to PVDF membranes. Next, the membranes were blocked with NcmBlot blocking buffer for 10 min in the ambient, with further trimming into as narrow strips according to the specific molecular weight of the protein of interest on the basis of the given markers. Later, the strips were incubated overnight at 4 °C with the appropriate primary antibodies: Caspase-3, HSP-70, HSP-90, AKT, and GAPDH, diluted to 1:2000. After being washed in Tris-buffered saline and Tween (TBS-T) for 3 times, certain stripes were incubated with secondary horseradish peroxidase (HRP)-labeled goat anti-rabbit IgG diluted 1:5000 for 1 h in the ambient. Protein bands were determined by transilluminator and imaging analysis system (Bio-Rad, USA) with NcmECL imaging while the densities of which were calculated by Quantity One software (Bio-Rad, USA).

### In vivo evaluation on the antitumor therapeutic performance of the SNP-Fe@MNs

All animal experiments were approved by the Laboratory Animal Welfare and Ethical Committee of Xian Jiaotong University (No. 2022-1606). The B16-bearing Balb/c nude mouse (6-week-old female) model was utilized for research on the in vivo antitumor efficiency of MNs. 80 × 10^4^ B16 cells in 100 µL PBS were injected on the midback of mice subcutaneously. On day 6, when the volume of the tumor reached around 100 mm^3^, full thickness wounds slightly wider to adhere to tumor body were surgically produced, with no tumor tissue being excised during the whole procedure. After surgical exposure, the wound was then penetrated and covered with different types of MNs. During the therapy, the patches were fixed with a transparent membrane dressing on the surface.

For the NIR (+) group, an 808 nm laser (1.50 W cm^−2^, 15 min) was applied after the utilization of the MN patches from days 6 to 8. During the three consecutive treatments, the ranges of temperature in the wound area were recorded by the infrared camera. The volume of melanoma is measured by Caliper daily and calculated as ${\text{V}}=\frac{{{\text{ab}}}^{2}}{2}$, where a and b represent the longest and shortest diameters. Meanwhile photographs were taken on day 6, 9, 14, 17 and 20. The weight of mice was recorded every other day, and the mortality remained 0% during the treatment. At the end of the therapy on day 20, the mice were photographed and then sacrificed. The tumor tissues were carefully dissected, weighed, and photographed, then sliced and evaluated by H&E, Ki67 immunohistochemical, and TUNEL staining assays. Besides, the main organs (skin, heart, lung, liver, spleen and kidney) were analyzed by H&E staining assays.

### In vitro angiogenic differentiation and proliferation of SNP-Fe@MNs

Angiogenic assays were carried out according to the manufacturer’s instructions, as Matrigel was diluted in 1640 medium with SCMC@MNs, SNP-Fe@MNs-NO(−), and SNP-Fe@MNs-NO(+), then added to a 96-well plate, and incubated at 37 °C for 4 h for solidification. Significantly, during the whole regeneration experimental period, a group of microneedles that released NO were set up to simulate the process of releasing NO in the later stage for wound repair, thus, MNs were applied 60 min after UV irradiation according to the previous NO release curve to avoid the burst of gas. HUVECs, which were previously cocultured with different groups of MNs for 5 days, were then detached by digestion with 0.25% trypsin. HUVECs (1.5 × 10^4^) were later seeded on the mixture of Matrigel, and the 96-well plate was then incubated at 37 °C with 5% CO_2_ and tube formation was observed at 30 min and optimum 4 h. Furthermore, HUVECs were cocultured with different MNs, and the CCK-8 assay was performed at the end of days 1 and 2. Subsequently, the migration ability of HUVECs were measured through scratch assay, as the cells were incubated with the medium extracts of different types of MNs, and captured at 18 h and 24 h. The migration ratio was analyzed by Image J and measured by the following equation:

$$\text{Migration ratio }\left({\%}\right)=\frac{100\times \left(\text{Initial scratch area}-\text{Final scratch area}\right)}{\text{Initial scratch area}}.$$

To further explore the mechanism of proliferation, a real-time quantitative reverse transcription polymerase chain reaction (RT-qPCR) assay was utilized to analyze the expression of angiogenic genes in HUVECs, which were VEGF, HIF-1a, and endothelial nitric oxides (eNOs). After being incubated with medium extracts of three types of MNs as described above in the 6-well plates for 5 days, HUVECs were harvested with the TRIzol reagent on day 6 after the medium was removed. Then 1 μg total RNA was extracted according to the instructions of the RevertAid first-strand cDNA synthesis kit for reverse transcription, and a 2× SYBR Green Pro Taq HS Premix qPCR kit was used for the reactants’ preparation. The related primer sequences were listed in Additional file 1: Table S1, which were synthesized by AUGCT (Beijing, China). RT-PCR analysis was performed using a Multicolor Real-Time PCR Detection System (iQ5, Bio-Rad, USA). Relative gene expressions of interest were determined using β-actin as the housekeeping gene for normalization by using the 2^−ΔΔCt^ method. Meanwhile, Western Blot analysis were conducted as the processes stated above. The primary antibodies were HIF-1a, VEGF, AKT, p-AKT and β-actin (all diluted 1:2000).

### In vivo skin tissue regeneration capability of SNP-Fe@MNs

All animal experiments were approved by the Laboratory Animal Welfare and Ethical Committee of Xian Jiaotong University (No. 2022-1606). The Sprague–Dawley (SD) rat (7–8 weeks old, female) was anesthetized with isoflurane, depilated, disinfected, and drilled a round 17-mm-diameter hole-shaped full-thickness cutaneous back defect. For avoiding the wound from stretching or shrinking, an annular silicone ring was sewn adhesive to the defect, then groups of 17.5-mm-diameter MN patches were therefore attached to cover the wound surface. Subsequently, these wounds were fixed by transparent membrane dressings that were wrapped with sterile gauze to prevent gnawing and photographed at days 0, 4, 8, 12, and 16. The certain degree of wound closure was fitted into composite image, analyzed by image J and measured by the following equation:

$$\text{Wound Closure Ratio }\left({\%}\right)=\frac{100\times \left(\text{Intial wound area}-\text{Final wound area}\right)}{\text{Intial wound area}}.$$

By the end of the research at day 16, the wound tissues were sliced for further wound healing analysis via H&E, Masson, and CD31 immunohistochemistry staining assays.

### Statistical analysis

All experiments involved in this paper were implemented at least twice with 3–8 replicates and the data were presented as the mean ± standard deviation (SD). Error bars were considered as the SD of the mean of each independent samples among one certain experiment. The values of *P < 0.05, **P < 0.01 and ***P < 0.001 were statistically significant. Statistical significance were evaluated by the unpaired two-tailed t-test by GraphPad Prism Software and Origin Software.

### Supplementary Information

**Additional file 1:** **Figure S1**. (a) Photographs of SNP-Fe mixtures before (left) and after (right) UV irradiation. (b) FTIR spectrum of SNP-Fe mixtures before and after UV irradiation. (c) XRD of SNP-Fe mixtures before and after UV irradiation. (d) The absorption spectrum of SNP-Fe2+ solution before and after UV irradiation. (e) The concentration of NO in groups before and after the irradiation of NIR. **Figure S2**. Images of skin after pressing with MNs for 1 min. **Figure S3**. Images indicating migration of B16 cells (stained with crystal violet, rightmost row) after coculturing with SNP-Fe@MNs-NIR(−)-NO(+), SNP-Fe@MNs-NIR(+)-NO(−) and SNP-Fe@MNs-NIR(+)-NO(+). **Figure S4**. Proposed mechanisms of SNP-Fe@MNs dominated apoptosis via caspase 3-dependent HSP-70/HSP-90/AKT mediated signaling pathways. HSP-70, that abundant in cancers, proved to inhibit apoptosis through caspase-dependent mechanism by suppressing c-Jun N-terminal kinase (JNK), caspase-independent mechanism by suppressing apoptosis-inducing factor (AIF) and via the interaction with death receptors. In consequence, the upregulated of caspase 3 and down-regulated HSP-70/HSP-90/AKT and Ki67 induced the inhibition of tumor growth [1–3]. **Figure S5**. The weight-curve of dissected tumor in groups of Control, SNP-Fe@MNs-NIR(−)-NO(+), SNP-Fe@MNs-NIR(+)-NO(−) and SNP-Fe@MNs-NIR(+)-NO(+), respectively (n = 5. * *P *< 0.05; ** *P *< 0.01; *** *P *< 0.0001). **Figure S6**. Body weight-curve of mice in groups of Control, SNP-Fe@MNs-NIR(−)-NO(+), SNP-Fe@MNs-NIR(+)-NO(−) and SNP-Fe@MNs-NIR(+)-NO(+), respectively (n = 5, *P* > 0.05). **Figure S7**. H&E staining of skin and major organs of mice. **Table S1**. Primer - sequence used in this experiment.

## Data Availability

The data used to support the findings of this study are available from the corresponding author upon request.
