# Supplementary material for: A novel multifunctional microneedle patch for synergistic photothermal- gas therapy against maxillofacial malignant melanoma and associated skin defects
Source: J Nanobiotechnology. 2024 Apr 23;22:199. doi: 10.1186/s12951-024-02409-4 (PMC11036725; doi:10.1186/s12951-024-02409-4)
Supplement: Supplementary file 1 — Additional file 1: Figure S1. (a) Photographs of SNP-Fe mixtures before (left) and after (right) UV irradiation. (b) FTIR spectrum of SNP-Fe mixtures before and after UV irradiation. (c) XRD of SNP-Fe mixtures before and after UV irradiation. (d) The absorption spectrum of SNP-Fe2+ solution before and after UV irradiation. (e) The concentration of NO in groups before and after the irradiation of NIR. Figure S2. Images of skin after pressing with MNs for 1 min. Figure S3. Images indicating migration of B16 cells (stained with crystal violet, rightmost row) after coculturing with SNP-Fe@MNs-NIR(−)-NO(+), SNP-Fe@MNs-NIR(+)-NO(−) and SNP-Fe@MNs-NIR(+)-NO(+). Figure S4. Proposed mechanisms of SNP-Fe@MNs dominated apoptosis via caspase 3-dependent HSP-70/HSP-90/AKT mediated signaling pathways. HSP-70, that abundant in cancers, proved to inhibit apoptosis through caspase-dependent mechanism by suppressing c-Jun N-terminal kinase (JNK), caspase-independent mechanism by suppressing apoptosis-inducing factor (AIF) and via the interaction with death receptors. In consequence, the upregulated of caspase 3 and down-regulated HSP-70/HSP-90/AKT and Ki67 induced the inhibition of tumor growth [1–3]. Figure S5. The weight-curve of dissected tumor in groups of Control, SNP-Fe@MNs-NIR(−)-NO(+), SNP-Fe@MNs-NIR(+)-NO(−) and SNP-Fe@MNs-NIR(+)-NO(+), respectively (n = 5. * P < 0.05; ** P < 0.01; *** P < 0.0001). Figure S6. Body weight-curve of mice in groups of Control, SNP-Fe@MNs-NIR(−)-NO(+), SNP-Fe@MNs-NIR(+)-NO(−) and SNP-Fe@MNs-NIR(+)-NO(+), respectively (n = 5, P > 0.05). Figure S7. H&E staining of skin and major organs of mice. Table S1. Primer - sequence used in this experiment. [file 12951_2024_2409_MOESM1_ESM.docx]

**Additional file 1**

A Novel Multifunctional Microneedle Patch for Synergistic Photothermal - Gas Therapy Against Maxillofacial Malignant Melanoma and Associated Skin Defects

Shaojie Dong^123#^, Yuwei Zhang^12#^, Yifei Zhang^12^, Yukun Mei^12^, Ahmadi Sina^12^, Rui Zou^12*^ and Lin Niu^123*^

1. Key Laboratory of Shaanxi Province for Craniofacial Precision Medicine Research, College of Stomatology, Xi’an Jiaotong University, Xi’an 710004, Shaanxi Province, China

2. Clinical Research Center of Shaanxi Province for Dental and Maxillofacial Diseases, Xi’an 710004, Shaanxi Province, China

3. Department of Prosthodontics, College of Stomatology, Xi’an Jiaotong University, Xi’an 710004, Shaanxi Province, China

# These authors contributed equally to this work.

* Corresponding authors.

Rui Zou, rainy@ xjtu.edu.cn; Lin Niu, niulin@xjtu.edu.cn;


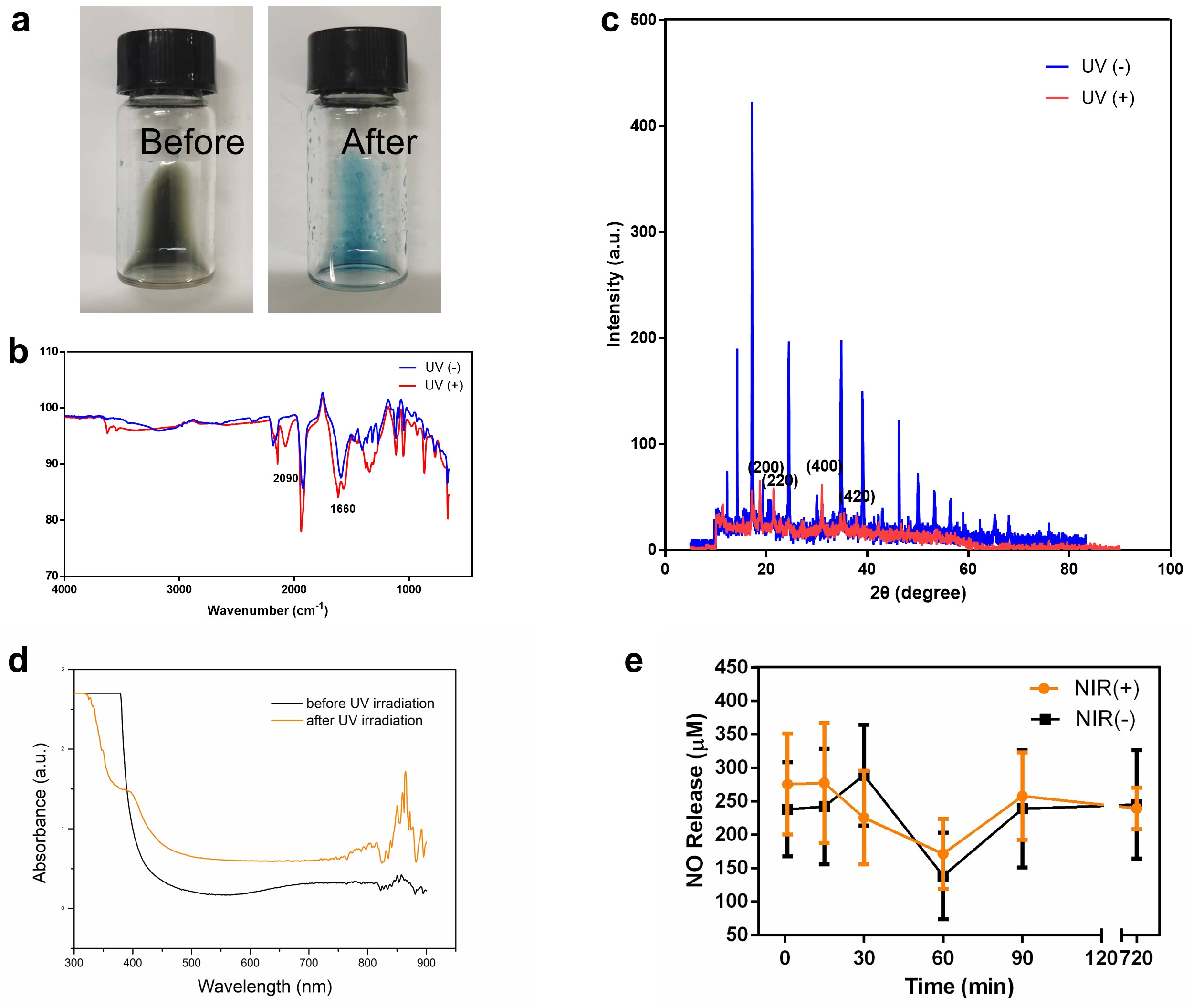


Figure S1. (a) Photographs of SNP-Fe mixtures before (left) and after (right) UV irradiation. (b) FTIR spectrum of SNP-Fe mixtures before and after UV irradiation. (c) XRD of SNP-Fe mixtures before and after UV irradiation. (d) The absorption spectrum of SNP-Fe2+ solution before and after UV irradiation. (e) The concentration of NO in groups before and after the irradiation of NIR.


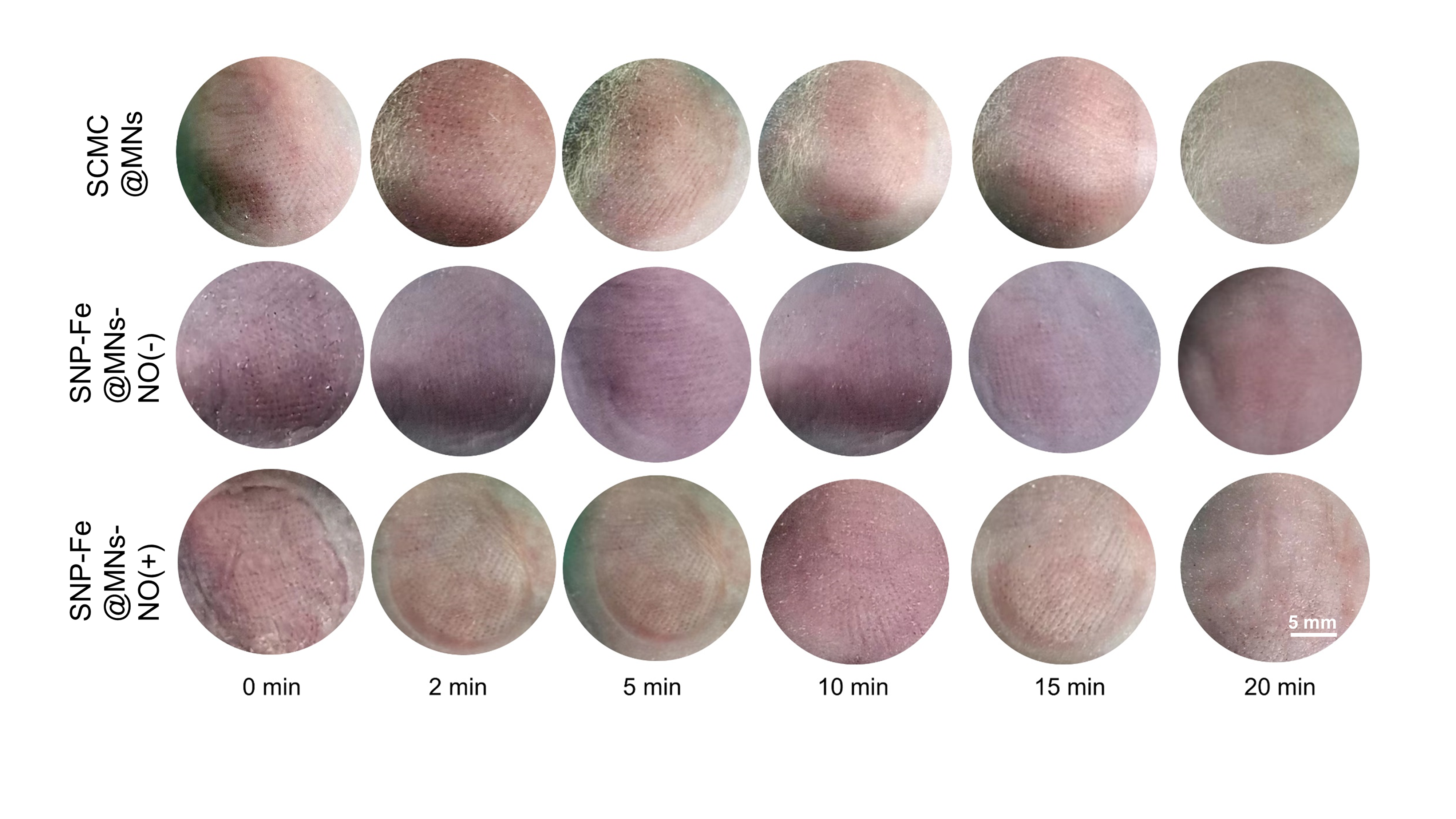


Figure S2. Images of skin after pressing with MNs for 1 min.


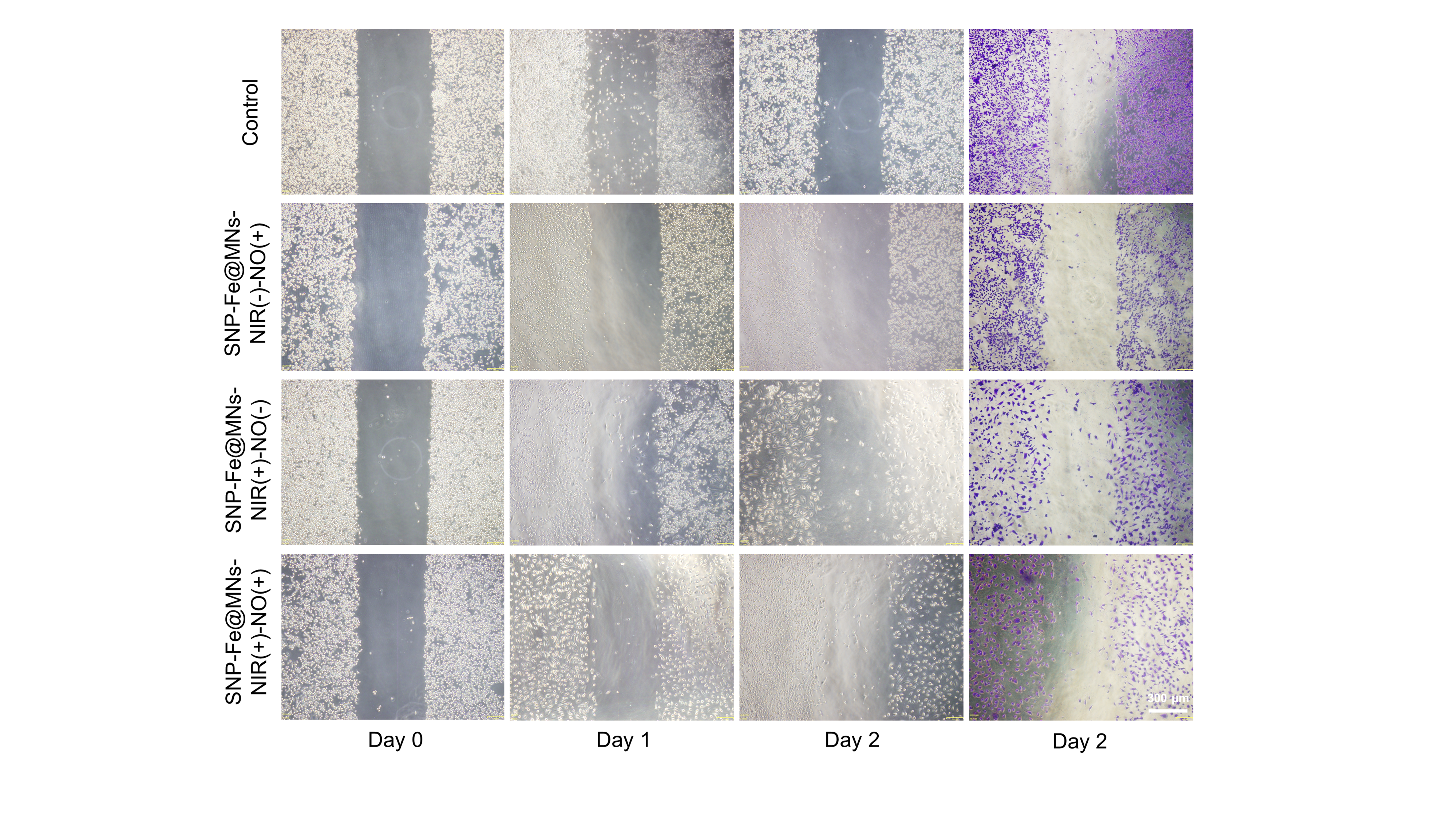


Figure S3. Images indicating migration of B16 cells (stained with crystal violet, rightmost row) after coculturing with SNP-Fe@MNs-NIR(-)-NO(+), SNP-Fe@MNs-NIR(+)-NO(-) and SNP-Fe@MNs-NIR(+)-NO(+).


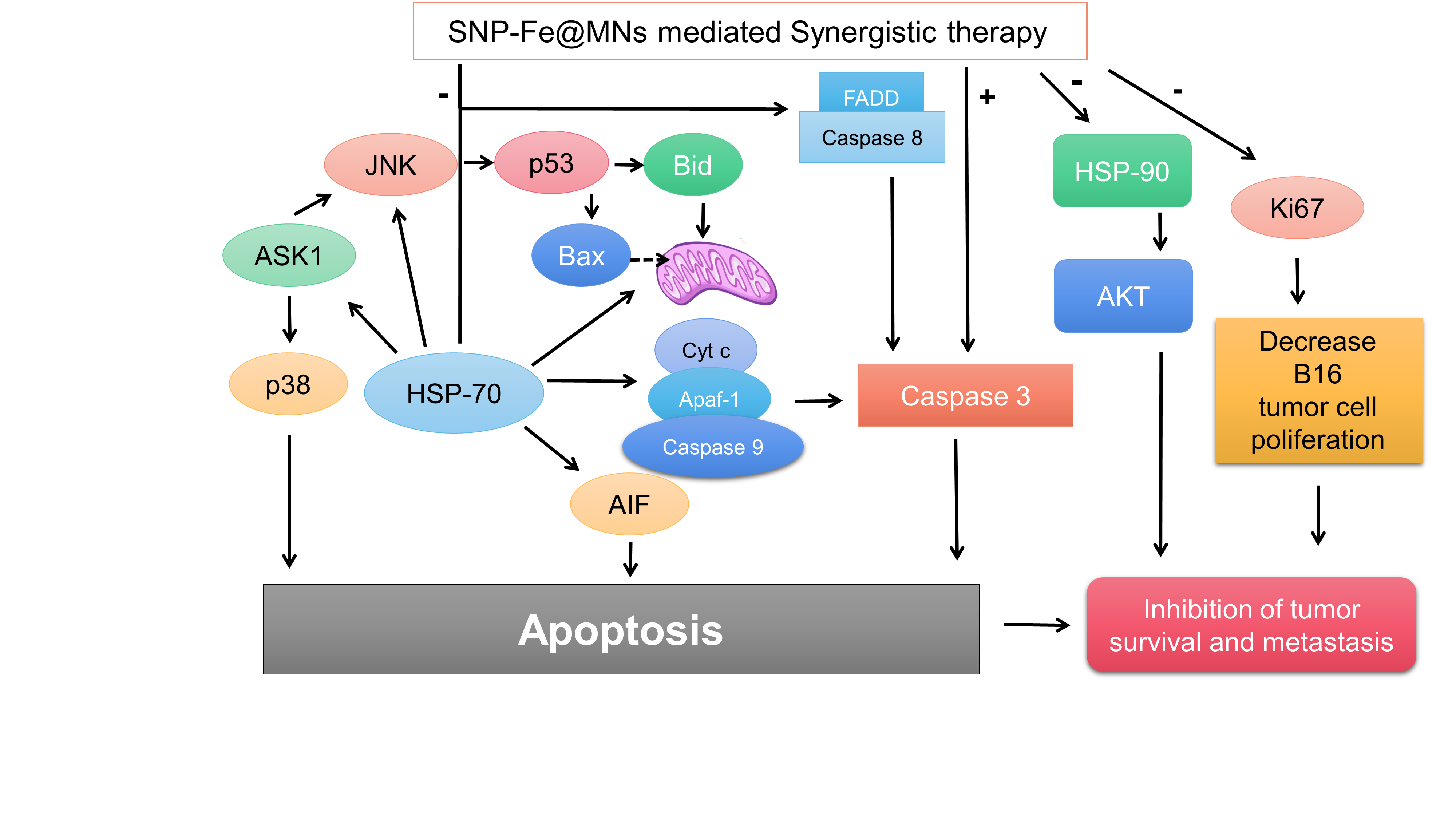


Figure S4. Proposed mechanisms of SNP-Fe@MNs dominated apoptosis via caspase 3-dependent HSP-70/HSP-90/AKT mediated signaling pathways. HSP-70, that abundant in cancers, proved to inhibit apoptosis through caspase-dependent mechanism by suppressing c-Jun N-terminal kinase (JNK), caspase-independent mechanism by suppressing apoptosis-inducing factor (AIF) and via the interaction with death receptors. In consequence, the upregulated of caspase 3 and down-regulated HSP-70/HSP-90/AKT and Ki67 induced the inhibition of tumor growth ^[1-3]^.


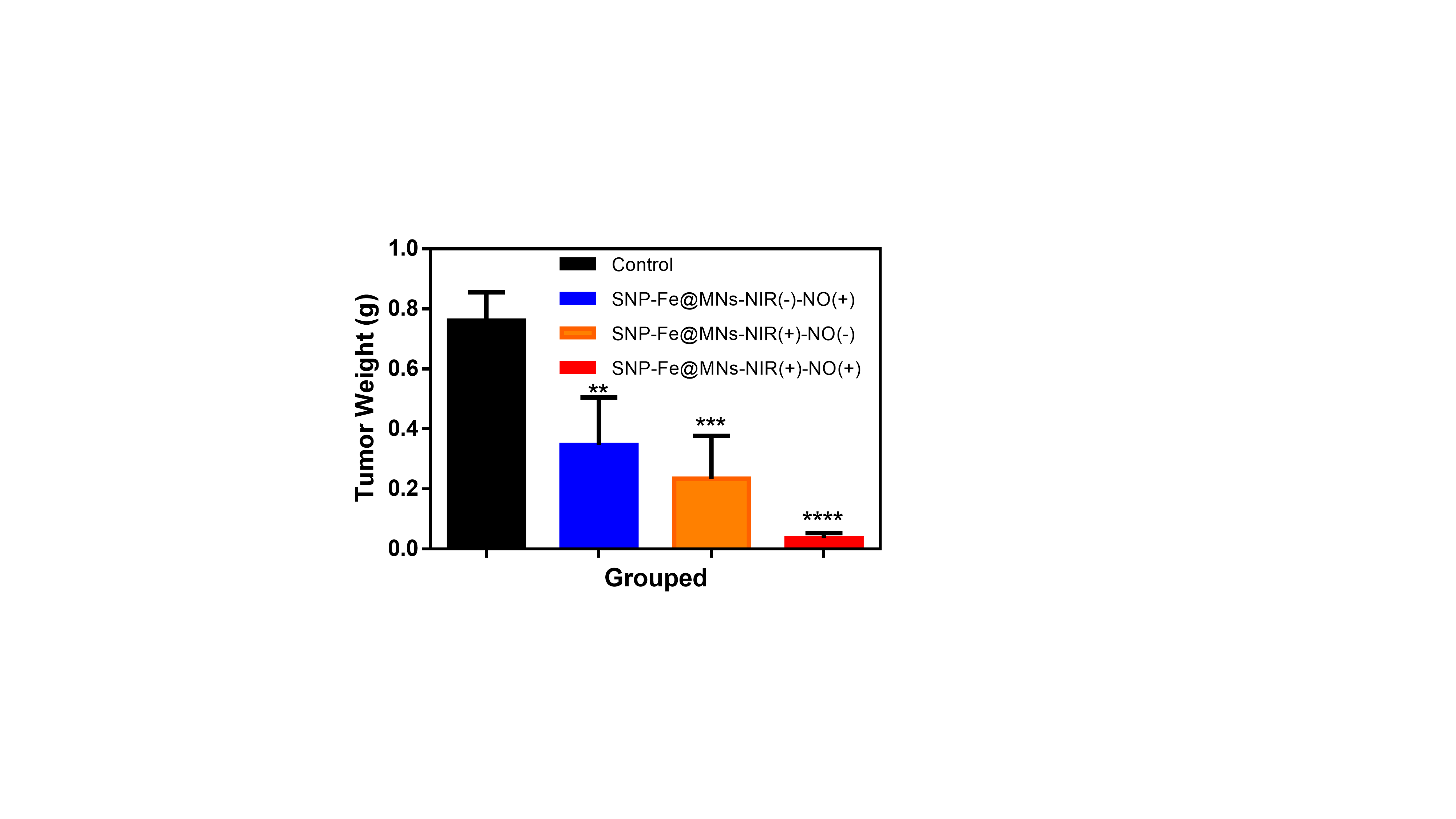


Figure S5. The weight-curve of dissected tumor in groups of Control, SNP-Fe@MNs-NIR(-)-NO(+), SNP-Fe@MNs-NIR(+)-NO(-) and SNP-Fe@MNs-NIR(+)-NO(+), respectively (n = 5. * *P* < 0.05; ** *P* < 0.01; *** *P* < 0.0001).


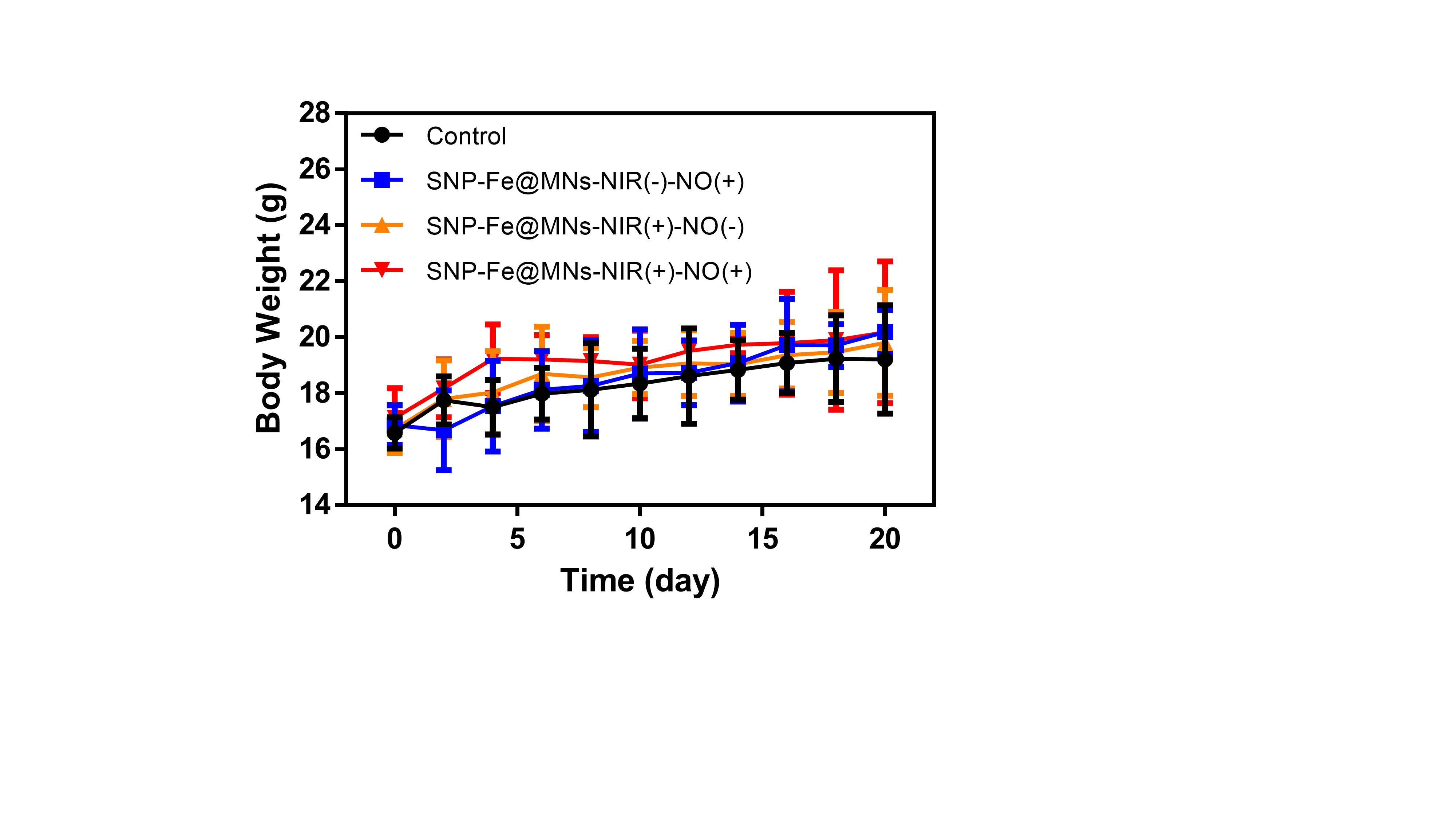
.

Figure S6. Body weight-curve of mice in groups of Control, SNP-Fe@MNs-NIR(-)-NO(+), SNP-Fe@MNs-NIR(+)-NO(-) and SNP-Fe@MNs-NIR(+)-NO(+), respectively (n = 5, *P* > 0.05).


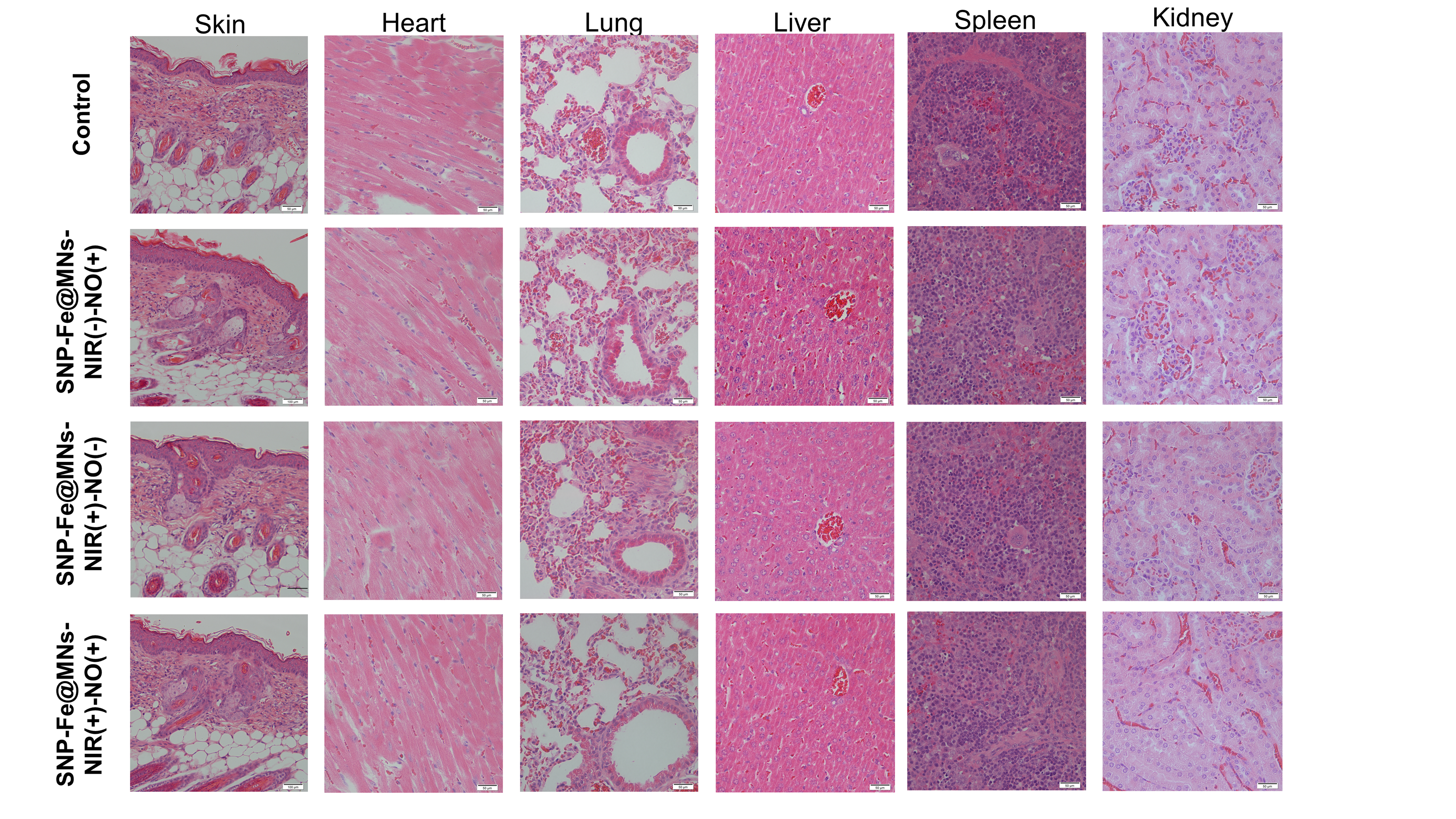


Figure S7. H&E staining of skin and major organs of mice.

Table S1. Primer - sequence used in this experiment.

| Primer | Sequence (5′−3′) |
| --- | --- |
| h VEGF | F 5′-TGCGGATCAAACCTCACCA  R 5′-CAGGGATTTTTCTTGTCTTGCT |
| h HIF-1a | F 5′-CCATGTGACCATGAGGAAAT  R 5′-CGGCTAGTTAGGGTACACTT |
| h eNOs | F 5′-TTCGGCTGCCACCTGATCCTAA  R 5′-AACATGTGTCCTTGCTCGAGGCA |
| h β-actin | F 5′-AGCGAGCATCCCCCAAAGTT  R 5′-GGGCACGAAGGCTCATCATT |

**References**

1. Albakova, Z., G.A. Armeev, L.M. Kanevskiy, E.I. Kovalenko, and A.M. Sapozhnikov, *HSP70 Multi-Functionality in Cancer.* Cells, **2020**. 9(3): 587

2. Hanahan, D. and R.A. Weinberg, *Hallmarks of cancer: the next generation.* Cell, **2011**. 144(5): 646-674

3. Shetake, N.G., A. Kumar, S. Gaikwad, P. Ray, S. Desai, R.S. Ningthoujam, et al., *Magnetic nanoparticle-mediated hyperthermia therapy induces tumour growth inhibition by apoptosis and Hsp90/AKT modulation.* Int J Hyperthermia, **2015**. 31(8): 909-919
